# Urbanization and humidity shape the intensity of influenza epidemics in U.S. cities

**DOI:** 10.1126/science.aat6030

**Published:** 2018-10-05

**Authors:** Benjamin D. Dalziel, Stephen Kissler, Julia R. Gog, Cecile Viboud, Ottar N. Bjørnstad, C. Jessica E. Metcalf, Bryan T. Grenfell

**Affiliations:** 1 Department of Integrative Biology, Oregon State University, Corvallis, OR, USA; 2 Department of Mathematics, Oregon State University, Corvallis, OR, USA; 3 Department of Applied Mathematics and Theoretical Physics, University of Cambridge, Cambridge, UK; 4 Division of International Epidemiology and Population Studies, Fogarty International Center, National Institutes of Health, Bethesda, MD, USA; 5 Department of Entomology, Pennsylvania State University, State College, PA, USA; 6 Department of Ecology and Evolutionary Biology, Princeton University, Princeton, NJ, USA; 7 Woodrow Wilson School of Public and International Affairs, Princeton University, Princeton, NJ, USA

## Abstract

Influenza epidemics vary in intensity from year to year, driven by climatic conditions and by viral antigenic evolution. However, important spatial variation remains unexplained. Here we show predictable differences in influenza incidence among cities, driven by population size and structure. Weekly incidence data from 603 cities in the United States reveal that epidemics in smaller cities are focused on shorter periods of the influenza season, whereas in larger cities, incidence is more diffuse. Base transmission potential estimated from city-level incidence data is positively correlated with population size and with spatiotemporal organization in population density, indicating a milder response to climate forcing in metropolises. This suggests that urban centers incubate critical chains of transmission outside of peak climatic conditions, altering the spatiotemporal geometry of herd immunity.

Predicting the epidemiology and evolution of influenza is an important goal for public health and an approaching milestone in the study of complex systems (*1*, *2*). Patterns of influenza spread and diversification are shaped by interacting ecological and evolutionary processes, including viral antigenic evolution (*3**–**6*), climatic conditions affecting transmission potential (*7*), and spatial heterogeneity in transmission among hosts, from local (*8*, *9*) to regional (*10*, *11*) to global scales (*12*). A global latitudinal gradient in epidemic periodicity (withmore strongly seasonal epidemics at temperate latitudes) is associatedwith climatic variation (*13*), with fluctuations in specific humidity as a key climatic driver (*14*). In temperate regions,multiyear “boom and bust” cycles in strain-specific incidence are associated with epochal evolution, involving intermittent jumps through antigenic space driven by antigenically localized susceptible depletion (*3*, *4*). This process is coupled to an evolutionary backbone shaped by global migration patterns in the virus (*15*), including repeated seeding from persistent regions, particularly in Asia (*16*, *17*). At regional scales, differences in epidemic timing are correlated with patterns of human contact, including commuting patterns and the timing of school terms (*10*, *11*, *18*, *19*). Robust epidemic and antigenic forecasts require a predictive understanding of the emergent properties of these interacting processes (*20*, *21*).

Cities are the principal locations for influenza transmission in humans (*22*), and therefore the primary context where drivers of transmission interact. However, recent comparisons of citylevel influenza transmission patterns have revealed unexplained differences among cities within the same broad climatic and antigenic regimes, suggesting that endogenous differences among cities may interact with climatic and evolutionary drivers to cause divergent epidemic dynamics at the city level (*23*, *24*). Cities can differ from each other in several ways that could potentially influence influenza transmission, including variation in the timing and coverage of public health interventions (*25*, *26*) and variation in population health and socioeconomic conditions (*27**–**29*). Cities also differ fundamentally in population size, spatial structure, and connectivity, in ways that may affect infectious contact patterns (*30*, *31*). These have the potential to substantially alter epidemic dynamics, including responses to climate forcing, and the impacts of public health interventions (*28*, *32*, *33*). However, the role of city size and structure in shaping transmission patterns of seasonal influenza is not well understood.

We address this here using 6 years (2002 to 2008) of data on weekly incidence of influenzalike illness (ILI) in 603 three-digit postal (ZIP) codes across the United States, assembled from medical claims data (*34*). ZIP codes are designed for efficient mail distribution such that the first three digits typically represent a contiguous geographic area surrounding amajor city. Incidence in a ZIP code is measured as the proportion of physician visits that are for ILI in a given week, and is strongly correlated with U.S. Centers for Disease Control and Prevention (CDC) reference influenza surveillance time series (Spearman’s ρ > 0.88). Our analysis corrects for sensitivity and specificity of ILI surveillance as an estimate of influenza incidence by incorporating cityspecific reporting rates that vary temporally between the peak and off-peak influenza season (materials and methods).

The ILI data show persistent differences among cities in how influenza incidence is distributed throughout each season (Fig. 1, A and B). Let the incidence distribution *p_ij_* represent the fraction of ILI incidence in influenza year *j* (centered on Northern Hemisphere winter: 1 July to 30 June) that occurred during week *i* (weeks from 1 July of the current influenza year) in a given city, and define epidemic intensity, v*j*, as the inverse of the Shannon entropy of incidence distribution in a given city and year, *v_j_* = ${\left(-\sum_{i}p_{ij}\log\;p_{ij}\right)}^{-1}$, which we normalize to be between 0 and 1 by subtracting the global minimum and then dividing by the global maximum across all cities. Epidemic intensity thus defined is minimized when incidence is spread evenly across weeks and increases as incidence becomes more intensively focused in particular weeks. Because v*j* is a function of incidence distribution, rather than raw incidence, it is invariant under differences in overall reporting rates and/or attack rates among cities and years.

**Fig. 1:**
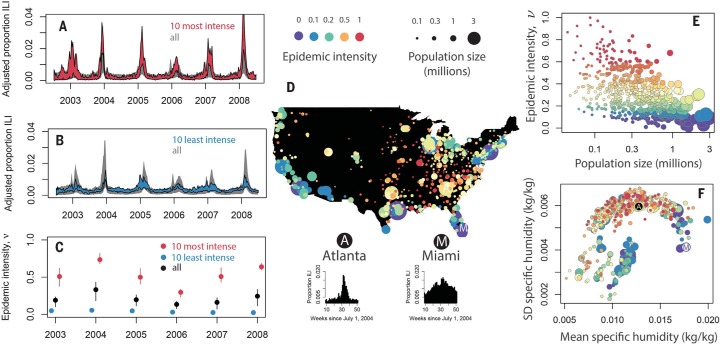
**Systematic differences among U.S. cities in the intensity of seasonal influenza epidemics.** (**A** to **C**) Differences among cities in epidemic intensity are preserved across years, indicated by comparing the temporal dynamics of the cities with the highest and lowest average intensity. Points show means, vertical lines show interquartile ranges, and polygons enclose the central 95% of ILI incidence data that have been corrected for intercity variation in background incidence and reporting, by linear transformation of each city’s time series to have minimum 0 and a common total attack rate over the 6-year period. (**D** to **F**) Cities with higher mean intensity tend to be located in the east, have smaller population sizes, and have higher-amplitude seasonal fluctuations in specific humidity. In (F), the vertical axis is standard deviation (SD) and the labeled points are Atlanta (A) and Miami (M).

We find that differences in v*j* among cities persist across years—some cities have consistently more intense epidemics than others, year after year. These differences among cities are epidemiologically significant, comparable in magnitude to differences in intensity among years associated with antigenic shifts [e.g., the increase in epidemic intensity across all cities in the 2003–2004 season associatedwith the A/Fujian/411/02 (H3N2) strain variant is comparable in magnitude to differences in intensity among cities in any season; Fig. 1C], and are also apparent in separate publicly available data, and across wider time scales, including since the 2009 pandemic (29) (fig. S1).

Differences inmean intensity $\propto \sum_{j}v_{j}$ Show a geographic pattern, with intense epidemics focused in the east (Fig. 1D).Mean intensity also varies with population size (Fig. 1E) and climate (Fig. 1F). In particular, ν tends to be higher in smaller populations, especially those with high amplitude in seasonal fluctuations of specific humidity.

We hypothesize that these patterns are caused by differential responses to climate forcing, mediated by divergent spatiotemporal patterns of transmission potential in cities of different sizes. By transmission potential, we mean the propensity for two randomly selected hosts in a population to attain spatiotemporal proximity sufficient for influenza transmission—sufficient proximity for the transfer of respiratory droplets from one host to the other. As specific humidity decreases in the winter, influenza virus remains viable outside a host for longer, expanding the spatiotemporal “cloud of risk” generated by an infected host and increasing transmission potential in the population. Seasonal, climate-driven increases in transmission potential thus drive the reproductive number of the infection (the expected number of secondary cases caused by an index case) upward in winter, eventually leading to an epidemic (*23*, *24*, *35*). However, climate is less important when the spatiotemporal distance between a pair of hosts is small, as is the case for a subset of potential contacts, such as those that reside, travel, or work in close proximity (fig. S2). This base transmission potential—transmission potential that is not strongly modulated by climate—could influence epidemic dynamics by facilitating influenza transmission over a wider range of climatic conditions, in turn reducing population- level susceptibility during the peak influenza season.

We thus propose that elevated base transmission potential in the presence of climate forcing leads to divergent epidemics among cities: Increased base transmission potential in urban centers enhances influenza spread outside of peak season, which elevates herd immunity to currently circulating strains, and subsequently attenuates explosive spread when climatic conditions are most favorable for transmission. This leads to the counterintuitive outcome that larger cities, with higher base transmission potentials, have more diffuse influenza epidemics. Base transmission potential may be elevated in large cities as a consequence of increased spatial organization, including aggregation of residences and workplaces, and the prevalence of high-density mass transit, among other factors (*30*, *31*).

We first demonstrate this effect using a standard climate-forced susceptible-exposedinfected- removed-susceptible (SEIRS) compartmental model for influenza epidemics (Fig. 2; materials and methods). Individuals enter the susceptible compartment in the model via immune waning following infection.New infections are generated by exposure of a susceptible individual to an infectious individual, at rate $\frac{\text{β}SI}{N}$ where N represents populations size, and *S*(*t*) and *I*(*t*) are functions of time that represent the number of susceptible and infectious individuals, respectively. For a given number of susceptible and infected individuals, the rate of appearance of new infections is governed by the transmission function β(t) = κ + σe^‒ω^*^q^*^(^*^t^*^)^, where κ represents city-level base transmission potential, σ the maximum gain in transmission potential at 0 specific humidity, and ω the rate of loss in viral viability due to specific humidity *q*(*t*), in units of kg/kg. The transmission function β(*t*) thus consists of a sum of two components: a seasonally invariant base transmission potential κ , representing transmission among contactswhose close spatiotemporal proximity renders climatic conditions moot; and additional transmission modulated by specific humidity, σe^‒ω^*^q^*^(^*^t^*^)^, which increases as drier conditions in U.S. cities in the winter increase the risk of transmission over larger spatiotemporal distances. This transmission function has been successfully used to fit and forecast seasonal influenza epidemics in previous studies (*14*, *24*).

**Fig. 2:**
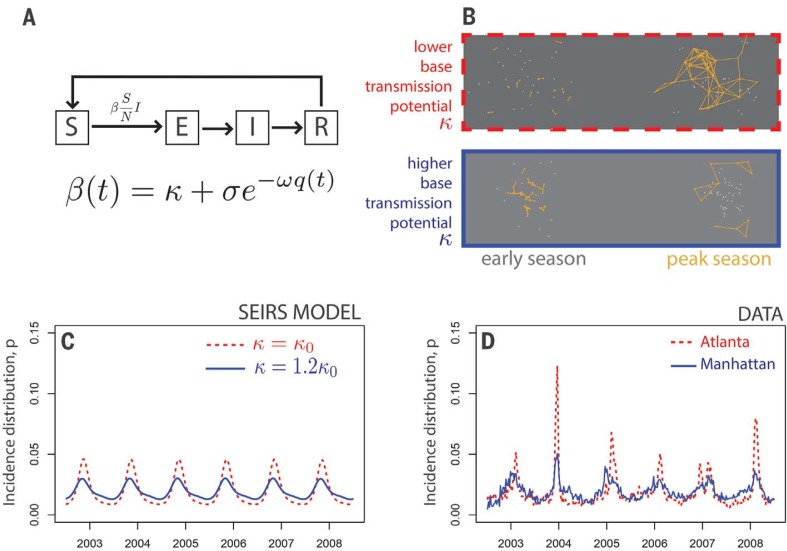
**Increasing base transmission potential can decrease epidemic intensity in a seasonally forced compartmental epidemic model**. (**A**) Diagram of a susceptible-exposedinfected- removed-susceptible (SEIRS) model. The seasonally varying transmission rate β depends on specific humidity *q* and the base transmission potential of the population, κ. See materials and methods for details. (**B**) Diagram of transmission in two hypothetical populations. Points represent individual hosts and yellow lines show transmission events. In populations with higher base connectivity, chains of transmission are longer during the early influenza season, when climatic conditions are not yet ideal for wider spread. (**C**) Simulations of the model for two levels of base transmission (red and blue lines), which yield corresponding variation in epidemic intensity. (**D**) Incidence distributions in U.S. three-digit ZIP codes (e.g., Atlanta and Manhattan) show comparable variation in epidemic intensity, and also evidence of seasonal variation in transmission rates and reporting, which are included in the model during fitting (Fig. 3; materials and methods).

The SEIRS model shown in Fig. 2 is proof of concept that increasing base transmission potential can decrease epidemic intensity, as predicted. However, there are several obstacles to confronting the model with incidence data in its current form. First, the model is a forced nonlinear oscillator, so small changes in parameter values may produce large changes inmodel predictions, which substantially complicatesmodel fitting. Second, whereas the model assumes that incidence is perfectly observed in continuous time, the data consist of discrete (weekly) observations in each city, affected by city-specific time-varying differences in reporting rates. Finally, the model does not include interyear variation in transmission rates due to antigenic evolution.

Following previous work (*35**–**37*), we constructed a time-series approximation of the SEIRS model to work with city-level ILI data that accounts for variation in observed incidence driven by reporting and antigenic evolution (materials and methods). The resulting city-level time series models had 11 fitted parameters per city, i.e., <2 per year of incidence data, yet produced a strong match with the data, via n-week ahead (1 < *n* < 303) epidemic simulations (hindcasts; Fig. 3, A to C; Spearman’s ρ = 0.93 for comparison of observed and model-predicted intensity). Out-of-sample simulation performance was similarly strong (fig. S3).However, randomly reassigning κ estimates to cities destroyed the correspondence between the simulations and the data (fig. S4). Assessing the performance of *n*-week ahead time-series simulations requires comparingmultiple features of the data andmodel predictions (*38*), and we also include time-series plots of observed and simulated data in each city in the supplementary materials.

**Fig. 3:**
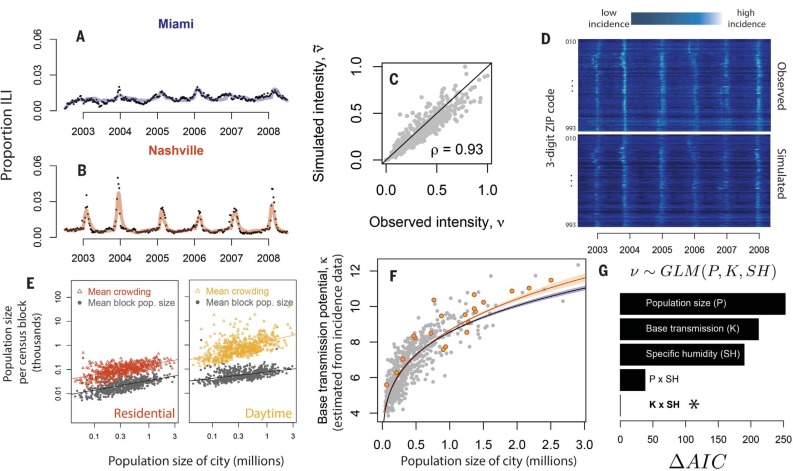
**Base transmission potential and specific humidity predict observed differences in the intensity of influenza epidemics across U.S. cities.** (**A** and **B**) *n*-step ahead simulation performance of the fitted SEIRS model, 1 < *n* < 303 weeks, in two cities. (**C**) Observed versus forward simulated average epidemic intensity in all cities. (**D**) Observed and simulated incidence in all cities and years. (**E**) Larger cities have more organized spatial population distributions and mobility patterns. Gray points show expected population size in a randomly selected census block in each city; colored points show expected block-level population size experienced by a randomly selected individual in each city [Lloyd’s mean crowding $\text{m}*=\bar{m}+\frac{\sigma_{\bar{m}}^{2}}{\bar{m}}-1$, where $\left(\bar{m}\right)$ represents mean population size in a census block, and $\sigma_{m}^{2}$ variance in population size across census blocks (31, 39)]. Mean crowding increases above mean block-level population size as spatial locations of individuals become more highly organized. (**F**) Population size and crowding estimated from census data predict base transmission potential estimated from ILI incidence data. Blue line shows fit for population size alone; yellow line, population size and crowding. Polygons enclose 1 SE around the fitted curves. Yellow points show the 20 cities with the most residential crowding. (**G**) Information-theoretic comparison of population size, climatic fluctuations, and fitted base transmission potential (i.e., base transmission potential predicted from population size and crowding rather than fitted to the incidence data) as predictors of observed epidemic intensity, via generalized linear models.

Fitting the model to ILI time-series data from each city reveals that differences in κ, interacting with local patterns in specific humidity, are sufficient to explain observed differences in epidemic intensity among cities (Fig. 3, C, E, and F). Base transmission potential is correlated with overall population size in a city (*N*), with the average population size of a census block $\left(\bar{m}\right)$, and with the level of crowding in each city (m*) (Fig. 3, E and F, and fig. S5). Crowding is measured as the expected blocklevel population size experienced by a randomly selected individual within a city, $\text{m}*=\bar{m}+\frac{\sigma_{m}^{2}}{\bar{m}}-1$ (*31*, *39*). As individuals within a city become aggregated within fewer focal locations, m* increases above $\bar{m}$. We find that both m* and $\bar{m}$ scale with city size (Fig. 3E), such that in large cities, residential and daytime population distribution are more highly organized. Moreover, circadian cycles of aggregation are more profound in large cities (steeper slope in daytime mean crowding compared to residential mean crowding as functions of population size in Fig. 3E: daytime slope = 0.537 ± 0.036, residential slope = 0.412 ± 0.023 SE).

Data on crowding substantially improve predictions of κ , relative to using population size alone, assessed using Akaike information criterion (AIC; ΔAIC = 37.36; Fig. 3F). Moreover, after adjusting for the effects of population size, residual crowding in cities is correlated with residual base transmission potential—cities that have more crowding for their size also have higher fitted values for k than expected for their size ( *p* < 0.0001 for linear regression of excess connectivity as a function of excess residential crowding and *p* = 0.03 for linear regression of excess connectivity as a function of excess daytime crowding)—consistent with the hypothesis that increased spatial organization in larger cities is driving increases in κ . Finally, interactions between κ and specific humidity provide a much stronger statistical fit to observed intercity variation in epidemic intensity, ν, relative to models featuring only specific humidity and/or population size (Fig. 3, D and G).

Regional correlations in seasonal influenza incidence have been linked with regional variation in city sizes and associated variation in intercity connectivity: All else being equal, random epidemic extinctions are less likely in large populations, and metropolises are more strongly interconnected by patterns of human travel, which synchronize epidemics among cities (*10*). However, influenza transmission dynamicswithin cities have generally been assumed to conform to the assumptions of mass action, precluding systematic intercity differences in epidemic dynamics that are driven endogenously by differential contact patterns. By contrast, our results show that processes underlying epidemic persistence and interconnectivity are rooted at the intracity scale and drive divergent, yet highly predictable, responses to climate forcing among cities of different sizes, which then scale up to influence regional epidemic patterns. Because large cities are also hubs in the intercity travel network, spatial aggregation of populations in large cities could be a proxy for the intensity of infectious contact both within and among cities. In this context, a key uncertainty is how external seeding of infections may drive epidemic patterns among cities of different sizes, and more generally, how transmission processes within cities drive patterns in epidemic intensity at different scales of observation (*29*).

Our model predicts that changes in urbanization and climate will lead to specific changes in the intensity of future influenza epidemics. In particular, increasing the amplitude of seasonal fluctuations in specific humidity leads to more intense epidemics in ourmodel; however, elevated base transmission potential in metropolises could counteract this effect (fig. S6). Notably, vaccination early in the season could mimic the accumulation of population-level immunity via off-peak transmission, increasing both direct and indirect protection (*40*) and regulating the intensity of seasonal epidemics; this illustrates an additional population-level benefit to influenza vaccination under increasingly extreme climate cycles. At the same time, state-level variation in vaccination coverage is not associated with variation in epidemic intensity across cities (fig. S8), perhaps because yearly variation in vaccine efficacy dwarfs geographic differences in vaccine coverage, obscuring any residual effect of vaccination on the spatial patterning of epidemics (*29*, *40*).

The scale of influenza epidemics can sometimes mirror that of pandemics—for example, the recent influenza seasonal outbreak in winter 2017–2018 had a similar epidemic size and peak intensity as that of the 2009 pandemic in the United States. More research is needed to understand and predict the scale and intensity of influenza outbreaks, as a function of population susceptibility and spatial organization, and the potential tradeoffs between these epidemic parameters. Our work indicates potential trade-offs between scale and intensity of epidemics that raise important questions for future work on the optimization of health systems against endemic and pandemic threats.

Increased epidemic intensity demands increased surge capacity in the public health system, including primary care facilities and clinical laboratories (*41*). This is particularly important for influenza, where the impact of vaccination depends on timely development and distribution of annual vaccines (*40*). Our analysis shows that some of the cities with the most intense influenza epidemics (driven by low base transmission potentials) are also among those with the most challenging socioeconomic conditions (fig. S7) (*27*). This is congruent with recent analyses of socioeconomic determinants of influenza mortality at the intracity level (*28*). Statistical associations between socioeconomic conditions and influenza dynamics may thus be caused in part by underlying variation in human aggregation patterns: For instance, metropolises have highly aggregated cores with high base transmission potentials, where median per-capita income is also higher. Our results also underscore the importance of considering spatial heterogeneity when assessing the impacts of climate forcing on infectious disease dynamics. As has recently been demonstrated for diarrheal diseases (*33*), spatial patterns in population density within cities can modulate the impact of climate variation on disease transmission patterns. By extending this result to include influenza, our findings indicate the potential for systematic effects of metropolises on climate-driven disease dynamics across a range of pathogens.

The ecological and evolutionary dynamics of influenza depend on the locations of “fertile ground” for transmission: places and times where critical chains of transmission incubate immigrating viral lineages (*5*, *42*). Our results show how metropolises play a disproportionately important role in this process, as epidemic foci, and as potential sentinel hubs, where epidemiological observatories could integrate local strain dynamics to predict larger-scale patterns (*4*, *43*, *44*). As the growth and form of cities affect their function as climate-driven incubators of infectious disease, it may be possible to design smarter cities that better control epidemics in the face of accelerating global change.

## Supplementary Material

Urbanization and humidity shape the intensity of influenza epidemics in U.S. cities

Urbanization and humidity shape the intensity of influenza epidemics in U.S. cities

## Data and materials availability

Data on epidemic intensity in each city and scripts for reproducing the statistical analyses are located at https://github.com/BenjaminDalziel/InfluenzaGeometry.

## SUPPLEMENTARY MATERIALS

www.sciencemag.org/content/362/6410/75/suppl/DC1

Data Files

References (*45*, *46*)

## References

[1] Gandon S., Day T., Metcalf C. J. E., Grenfell B. T., *Trends Ecol. Evol*. 31, 776–788 (2016). 27567404 10.1016/j.tree.2016.07.010

[2] Morris D. H. et al, *Trends Microbiol*. 26, 102–118 (2018). 29097090 10.1016/j.tim.2017.09.004 PMC5830126

[3] Koelle K., Cobey S., Grenfell B., Pascual M., *Science* 314, 1898–1903 (2006). 17185596 10.1126/science.1132745

[4] Łuksza M., Lässig M., *Nature* 507, 57–61 (2014). 24572367 10.1038/nature13087

[5] Zinder D. et al, *BMC Evol. Biol.* 14, 272 (2014). 25539729 10.1186/s12862-014-0272-2 PMC4316805

[6] Bedford T. et al, *Nature* 523, 217–220 (2015). 26053121 10.1038/nature14460 PMC4499780

[7] Lipsitch M., Viboud C., *Proc. Natl. Acad. Sci. U.S.A.* 106, 3645–3646 (2009). 19276125 10.1073/pnas.0900933106 PMC2656132

[8] Cauchemez S. et al, *Proc. Natl. Acad. Sci. U.S.A.* 108, 2825–2830 (2011). 21282645 10.1073/pnas.1008895108 PMC3041067

[9] Bourouiba L., Dehandschoewercker E., *J. W. M. Bush, J. Fluid Mech.* 745, 537–563 (2014).

[10] Viboud C. et al, *Science* 312, 447–451 (2006). 16574822 10.1126/science.1125237

[11] Charu V. et al, *PLOS Comput. Biol*. 13, e1005382 (2017). 28187123 10.1371/journal.pcbi.1005382 PMC5349690

[12] Tizzoni M. et al, *BMC Med.* 10, 165 (2012). 23237460 10.1186/1741-7015-10-165 PMC3585792

[13] Tamerius J. D. et al, *PLOS Pathog*. 9, e1003194 (2013). 23505366 10.1371/journal.ppat.1003194 PMC3591336

[14] Shaman J., Kohn M.*, Proc. Natl. Acad. Sci. U.S.A.* 106, 3243–3248 (2009). 19204283 10.1073/pnas.0806852106 PMC2651255

[15] Bedford T., Cobey S., Beerli P., Pascual M., *PLOS Pathog.* 6, e1000918 (2010). 20523898 10.1371/journal.ppat.1000918 PMC2877742

[16] Russell C. A. et al, *Science* 320, 340–346 (2008). 18420927 10.1126/science.1154137

[17] Wen F., Bedford T., Cobey S.*, Proc. Biol. Sci.* 283, 20161312 (2016). 27629034 10.1098/rspb.2016.1312 PMC5031657

[18] Cauchemez S., Valleron A.-J., Boëlle P.-Y., Flahault A., Ferguson N. M., *Nature* 452, 750–754 (2008). 18401408 10.1038/nature06732

[19] Gog J. R. et al, *PLOS Comput. Biol.* 10, e1003635 (2014). 24921923 10.1371/journal.pcbi.1003635 PMC4055284

[20] Chattopadhyay I., Kiciman E., Elliott J. W., Shaman J. L., Rzhetsky A., *eLife* 7, e30756 (2018). 29485041 10.7554/eLife.30756 PMC5864297

[21] Pei S., Kandula S., Yang W., Shaman J.*, Proc. Natl. Acad. Sci. U.S.A.* 115, 2752–2757 (2018). 29483256 10.1073/pnas.1708856115 PMC5856508

[22] United Nations, Department of Economic and Social Affairs, Population Division, “World Urbanization Prospects” (2014), pp. 1–32.

[23] Axelsen J. B., Yaari R., Grenfell B. T., Stone L., *Proc. Natl. Acad. Sci. U.S.A.* 111, 9538–9542 (2014). 24979763 10.1073/pnas.1321656111 PMC4084473

[24] Yang W., Lipsitch M., Shaman J., *Proc. Natl. Acad. Sci. U.S.A*. 112, 2723–2728 (2015). 25730851 10.1073/pnas.1415012112 PMC4352784

[25] Hatchett R. J., Mecher C. E., Lipsitch M., *Proc. Natl. Acad. Sci. U.S.A*. 104, 7582–7587 (2007). 17416679 10.1073/pnas.0610941104 PMC1849867

[26] Galarce E. M., Minsky S., Viswanath K., *Vaccine* 29, 5284–5289 (2011). 21621577 10.1016/j.vaccine.2011.05.014

[27] Chetty R. et al, *JAMA* 315, 1750–1766 (2016). 27063997 10.1001/jama.2016.4226 PMC4866586

[28] Grantz K. H. et al, *Proc. Natl. Acad. Sci. U.S.A.* 113, 13839–13844 (2016). 27872284 10.1073/pnas.1612838113 PMC5137773

[29] Lee E. C. et al, *PLOS Comput. Biol*. 14, e1006020 (2018). 29513661 10.1371/journal.pcbi.1006020 PMC5858836

[30] Bettencourt L. M. A., Lobo J., Helbing D., Kühnert C., West G. B., *Proc. Natl. Acad. Sci. U.S.A.* 104, 7301–7306 (2007). 17438298 10.1073/pnas.0610172104 PMC1852329

[31] Dalziel B. D., Pourbohloul B., Ellner S. P., *Proc. Biol. Sci.* 280, 20130763 (2013). 23864593 10.1098/rspb.2013.0763 PMC3730584

[32] Dalziel B. D. et al, *PLOS Pathog.* 10, e1004455 (2014). 25340642 10.1371/journal.ppat.1004455 PMC4207809

[33] Martinez P. P., King A. A., Yunus M., Faruque A. S. G., Pascual M., *Proc. Natl. Acad. Sci. U.S.A.* 113, 4092–4097 (2016). 27035949 10.1073/pnas.1518977113 PMC4839397

[34] Viboud C. et al, *PLOS ONE* 9, e102429 (2014). 25072598 10.1371/journal.pone.0102429 PMC4114744

[35] te Beest D. E., van Boven M., Hooiveld M., van den Dool C., Wallinga J., *Am. J. Epidemiol*. 178, 1469–1477 (2013). 24029683 10.1093/aje/kwt132

[36] Bjørnstad O. N., Finkenstadt B. F., Grenfell B. T., *Ecol. Monogr*. 72, 169–184 (2002).

[37] Dalziel B. D. et al, *PLOS Comput. Biol.* 12, e1004655 (2016). 26845437 10.1371/journal.pcbi.1004655 PMC4741526

[38] Held L., Meyer S., *J. Bracher, Stat. Med*. 36, 3443–3460 (2017). 10.1002/sim.7363 28656694

[39] Lloyd M., *J. Anim. Ecol*. 36, 1–30 (1967).

[40] Arinaminpathy N. et al, *Am. J. Epidemiol.* 186, 92–100 (2017). 28369163 10.1093/aje/kwx037 PMC5860220

[41] Crawford J. M. et al, *Emerg. Infect. Dis.* 16, 8–13 (2010). 20031036 10.3201/eid1601.091167 PMC2874380

[42] Hill N. J. et al, *Emerg. Infect. Dis*. 23, 654–657 (2017). 28322698 10.3201/eid2304.161668 PMC5367406

[43] Metcalf C. J. E. et al, *Lancet* 388, 728–730 (2016). 27059886 10.1016/S0140-6736(16)30164-7 PMC5678936

[44] Xue K. S. et al, *eLife* 6, e26875 (2017). 28653624 10.7554/eLife.26875 PMC5487208

