# Supplementary material for: Urbanization and humidity shape the intensity of influenza epidemics in U.S. cities
Source: Science. 2018 Oct 5;362(6410):75–9. doi: 10.1126/science.aat6030 (PMC6510303; doi:10.1126/science.aat6030)
Supplement: Urbanization and humidity shape the intensity of influenza epidemics in U.S. cities [file Science-362-75-s001.pdf]

Correction; see below

www.sciencemag.org/content/362/6410/75/suppl/DC1

Supplementary Materials for

Urbanization and humidity shape the intensity of influenza epidemics in U.S. cities

Benjamin D. Dalziel*, Stephen Kissler, Julia R. Gog, Cecile Viboud, Ottar N. Bjørnstad, C. Jessica E. Metcalf, Bryan T. Grenfell

*Corresponding author. Email: benjamin.dalziel@oregonstate.edu

Published 5 October 2018, *Science* **362**, 75 (2018)
DOI: 10.1126/science.aat6030

This PDF file includes:

Materials and Methods
Figs. S1 to S10
Table S1
References

Other Supplementary Materials for this manuscript include the following:
(available at www.sciencemag.org/content/362/6410/75/suppl/DC1)

Data Files (zipped archive)

Correction
The legend for fig. S2 listed the “hours elapsed” as ranging from 1 to 6, when in fact they ranged from 0.08 to 23.

Materials and Methods:

Data

We used weekly temperature, relative humidity and atmospheric pressure data for each city from Dark Sky (https://darksky.net), to calculate specific humidity, via the R package 'humidity' (https://cran.r-project.org/web/packages/humidity/index.html). Population sizes and Longitudinal Employer-Household Dynamics Origin-Destination Employment Statistics (LODES) commuting data are from the US Census (www.census.gov). Socioeconomic data are from (*27*). Vaccination coverage data are from US Centers for Disease Control and Prevention (CDC; www.cdc.gov/flu/fluvaxview).

SEIRS Model

The SEIRS model shown in Figure 2 is given by the following system of ordinary differential equations: $dS/dt = -N^{-1}\beta SI + \delta(N - S - E - I)$, $dE/dt = N^{-1}\beta SI - \varepsilon E$, $dI/dt = \varepsilon E - \gamma I$, where $\delta = 1/52$ weeks^{-1} is the immune waning rate, ε=7 weeks^{-1} is the rate of becoming infected following exposure (i.e. the expected amount of time that an individual spends in the exposed class is 1/7 weeks = 1 day), and γ=7/2 weeks^{-1} is the rate of recovery from infection (i.e the expected duration of infectiousness is 2 days), yielding a mean generation time of 3 days. In Figure 2C, κ_0=4, σ=4, and ω=40. Specific humidity was simulated as $q(t) = A \cos(2\pi (t - 5)/52) + B$, which closely matches the data for the US (fig. S9). Figure 2C uses values of A = 0.0086, B=0.012 for the lower κ (red curve) and A = 0.0068, B = 0.010 for the higher κ (blue curve), approximating specific humidity cycles in Atlanta and Manhattan respectively (fig. S9). We integrated the model forward for six years from initial conditions of 68% susceptible and 1 infected individual, to remove transient dynamics, then display a further six years of time evolution.

Fitting the model to the ILI data

Let I_{ij} represent the number infected in week *i* of season *j*. We calculated observed I_{ij} from the ILI incidence data by multiplying the incidence in each week by city population size, *N*, and rounding up to the nearest integer value.

Let S_{ij} represent the number susceptible in week *i* of season *j*, which is unobserved. We estimate S_{ij} from incidence data by assuming that the number of individuals who are susceptible declines monotonically over each season, proportional to cumulative incidence (*35*). Representing cumulative incidence by, $P_{ij} = \frac{1}{N}\sum_{k=0}^{i} I_{kj}$ and letting S_{0j} represent the unobserved initial number susceptible yields $S_{ij} = S_{0j}\exp(-\rho_i P_{ij})$, where the reporting parameter ρ_i describes the time-varying rate which transmission decreases with increasing cumulative incidence each season, dynamically adjusting for false positives and unreported cases (*35*). When fitting the model, we constrain ρ to have to alternate between two city-specific constants, one for peak influenza season, and one for the rest of the year (see below). The expected initial number susceptible is set to S_{0j}=0.68N, taken from (*24*).

The time-series model is given by $\mathrm{E}[I_{i+1,j}] = \beta_{ij}\frac{S_{ij}}{N}I_{ij}^{\alpha}$, where *E[]* represents the expected value of a random variable. , and $\beta_{ij} = \kappa + \sigma_j e^{-\omega q_{ij}}$ represents the discretized version of the

transmission function with specific humidity q_{ij} and season-specific transmission gain σ_j. Variation in σ is driven by strain differences in transmissibility from year to year.

The exponent α that appears in the time-series model is not part of the transmission function, but rather corrects for distortions resulting from discretizing the underlying continuous time branching process of contagious spread (*36*, *37*). These distortions scale with the number of cases, so by design α affects large numbers of cases more severely, but it does so in the same way both within and across cities. We set α=0.8 for all cities, to allow comparison of the transmission parameters across cities.

We confirmed that the observed scaling of κ with population size is not an artifact of α's impact on cities of different sizes by also fitting the time-series model to the incidence data for each city while holding population size constant, verifying that the positive relationship between actual population size and base transmission is preserved. We also note that base transmission is a much more powerful predictor of epidemic intensity than population size alone (Figure 3G), which suggests it is not an artifact of fitting the model to populations of different sizes.

Substituting the susceptible dynamics into the time series equation and log transforming produces

$$log\ E[I_{i+1,j}] = log\ (\kappa + \sigma_j\ exp(-\omega q_{ij})\ ) + log\ S_{0j} - \rho_i P_{ij} - log\ N + \alpha\ log\ I_{ij} \quad (1)$$

which we approximate by a piecewise linear function

$$log\ E[I_{i+1,j}] = log\ \kappa - \omega_1 q_{ij} + log\ S_{0j} - \rho_1 P_{ij} - log\ N + \alpha\ log\ I_{ij}, \quad q\ large\ (off\text{-}peak\ season)$$

$$log\ E[I_{i+1,j}] = log\ (\kappa+\sigma_j) - \omega_2 q_{ij} + log\ S_{0j} - \rho_2 P_{ij} - log\ N + \alpha\ log\ I_{ij}, \quad q\ small\ (peak\ season) \quad (2)$$

that becomes exact for $q=0$, and as q becomes large (fig. S10).

Whereas the transmission and reporting terms term in equation (1) are nonlinear, the approximation is linearized and can thus be fitted to data using robust techniques (i.e. generalized linear models). Importantly this approximation yields a good correspondence between model and data under forward simulation (Fig 3C,D, fig. S3). The two pieces of the function represent off-peak and peak season for influenza transmission respectively, as reporting rates and seasonal transmission gains (e.g. due to antigenic shifts) vary concurrently with specific humidity.

The corresponding generalized linear model has the form:

$$Y_{i+1,j} = a \cdot W_{ij} + b \cdot X_{ij} q_{ij} + c \cdot X_{ij} P_{ij} + O_{ij} + Z_{ij} \quad (3)$$

where $Y_{ij} = log[I_{ij}]$, a is a parameter vector with seven elements, $a = [log(\kappa), log(\kappa+\sigma_1), \ldots, log(\kappa+\sigma_6)]$, W_{ij} is a design vector with seven elements which indicates whether the data point associated with (i,j) is in the off-peak regime, or in one of the six influenza seasons in the data. The parameter vectors b and c are given by $b = [\omega_1, \omega_2]$, $c = [\rho_1, \rho_2]$ and the two-element design vector X_{ij} indicates whether the point associated with (i,j) is in the off-peak or peak influenza season.

The model contains an offset term $O_{ij} = log(<S_{0j}>) - log(N) + \alpha Y_{ij}$, where $<S_{0j}> = 0.68N$ represents the expected population-level initial susceptibility each season, taken from (*24*). The error term $Z_{ij} = D_j + \xi_{ij}$ captures noise, as well as latent seasonal variation in initial susceptibility $D_j = log(S_{0j}) - log(<S_{0j}>)$. $E[D_j] = 0$ and $E[\xi_{ij}]=0$ by definition, so $E[Z_{ij}] = 0$. Adapting the approach of (*35*), we define the peak influenza season to extend from 5 weeks before, to 5 weeks after the peak observed incidence each season.

Comparing statistical models of epidemic intensity

AIC scores shown in Figure 3G are the results of fitting Generalized Linear Models (GLM) of the form $log(v) \sim X*<q>*\sigma_q + <q>^2 + \sigma_q^2$ where v is our Shannon-diversity measure of epidemic intensity derived from the ILI time series, X is either $log(\kappa)$ or $log(N)$, $<q>$ represents mean specific humidity in kg/kg, σ_q is the standard deviation in specific humidity, and the notation $A*B$ for two terms means their main effects plus two-way interactions. An identity function was used as the link function. Instead of using κ fitted from incidence data, here we use the predicted value of κ from a fit to population size and crowding (Figure 3F), so that explanatory variables (κ, N) are derived from separate datasets on demography and climate and are entirely independent from the outcome of interest (ILI epidemic intensity). The GLMs were compared using differences in Akaike Information Criteria (ΔAIC), where larger values indicate models with poorer relative support, and ΔAIC>4 indicates weak relative support.

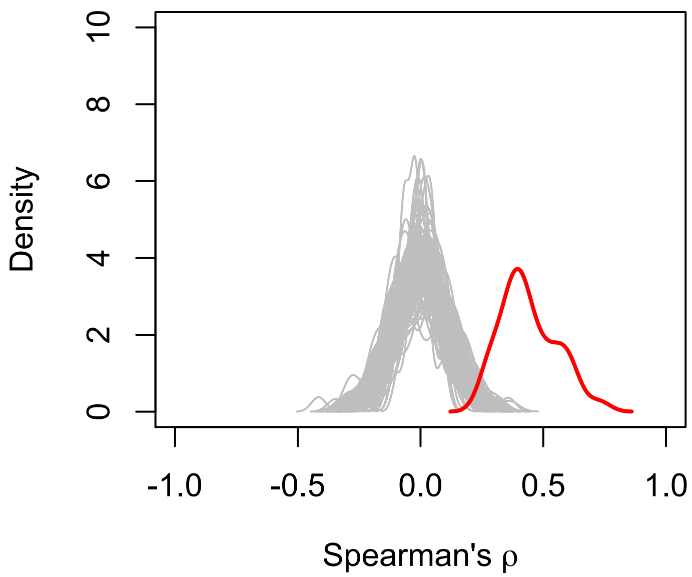

Figure S1. Correlations in epidemic intensity between years in 97 cities in the United States. Data are from Google Flu Trends (https://www.google.org/flutrends/about/data/flu/us/data.txt). The red line shows the empirical probability density. Grey lines show the distribution for 100 null simulations, where intensities in each season were randomly shuffled among cities. Epidemic intensities in cities are more correlated across years than would be expected by chance.

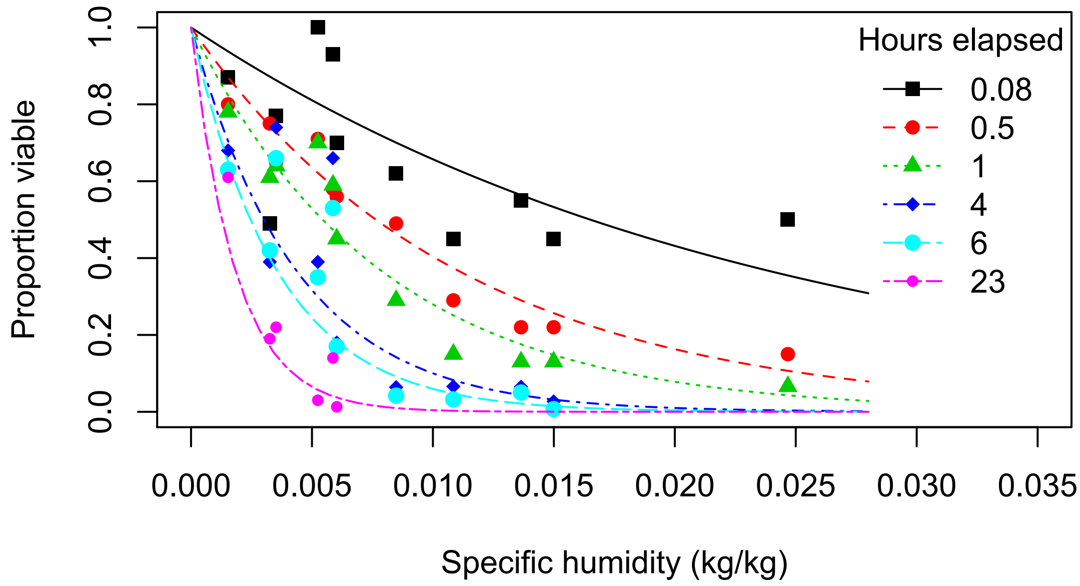

Figure S2. The rate of loss of viral viability due to specific humidity depends nonlinearly on the time elapsed. Assuming a diffusive process, as the spatiotemporal distance between shedding and uptake decreases, the impact of specific humidity becomes less profound. Points show data from (*45*). Lines show the function $f(q) = exp(-\omega(h)\ q)$ where $\omega(h)$ is the specific loss rate due to specific humidity observed after *h* hours, fitted by generalized linear models (on a log scale with an identity link function.) From h=0.08 to h=23 hours elapsed, the fitted values for ω(h) are: 42.00, 90.73, 127.48, 230.21, 282.31, 547.10.

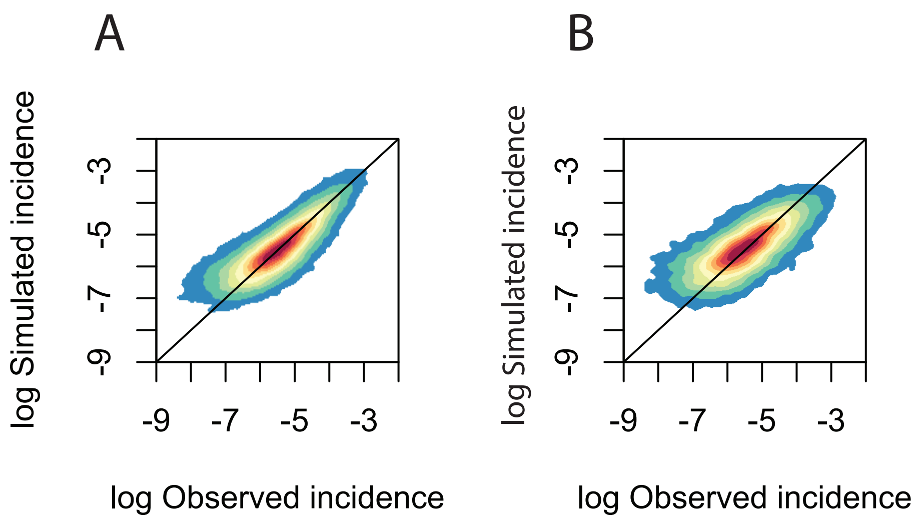

Figure S3. Scatter density of observed versus simulated ILI across all cities, with higher density indicated with warmer colors. (A) Using parameters estimated from the full six years of data in each city to predict the full six years. **(B)** Out-of-sample: simulating the final two years of data using parameters fitted from the first four years. In this case, values for σ_5 and σ_6 are taken as the mean of the fitted values over the previous four years.

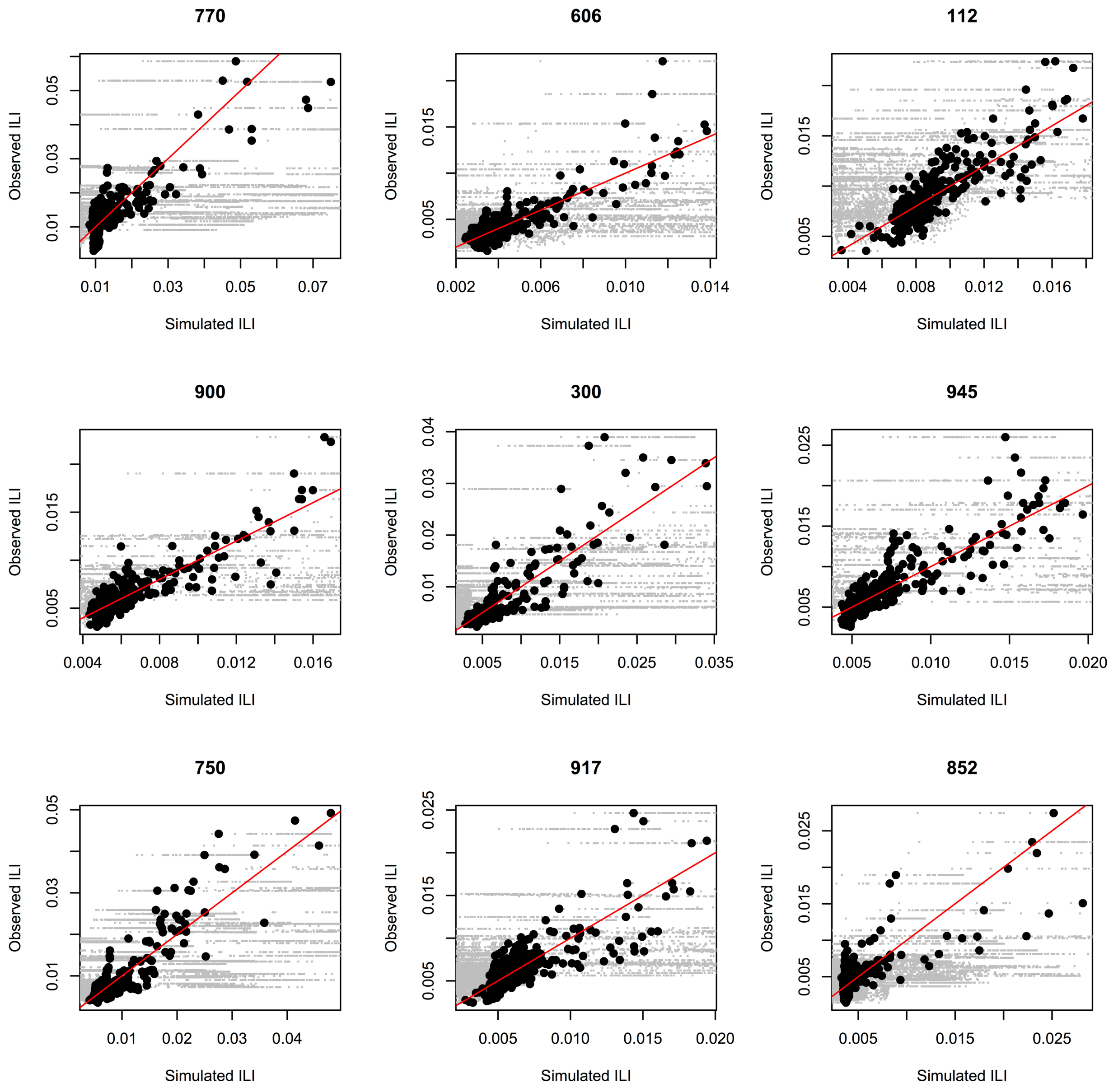

Figure S4. Observed and simulated ILI incidence in the nine largest cities in the data, labelled by their three-digit ZIP codes. Black points show forward simulations using the fitted parameters. Gray points show 100 replicate simulations using κ's randomly chosen from other cities.

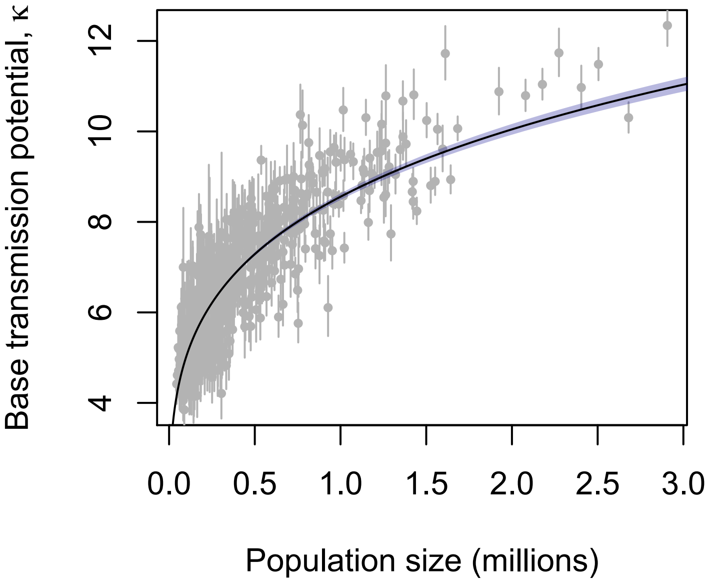

Figure S5. As in Figure 3F but showing uncertainty in estimates of κ within each city. Vertical lines span two standard errors in the estimate of κ for each city.

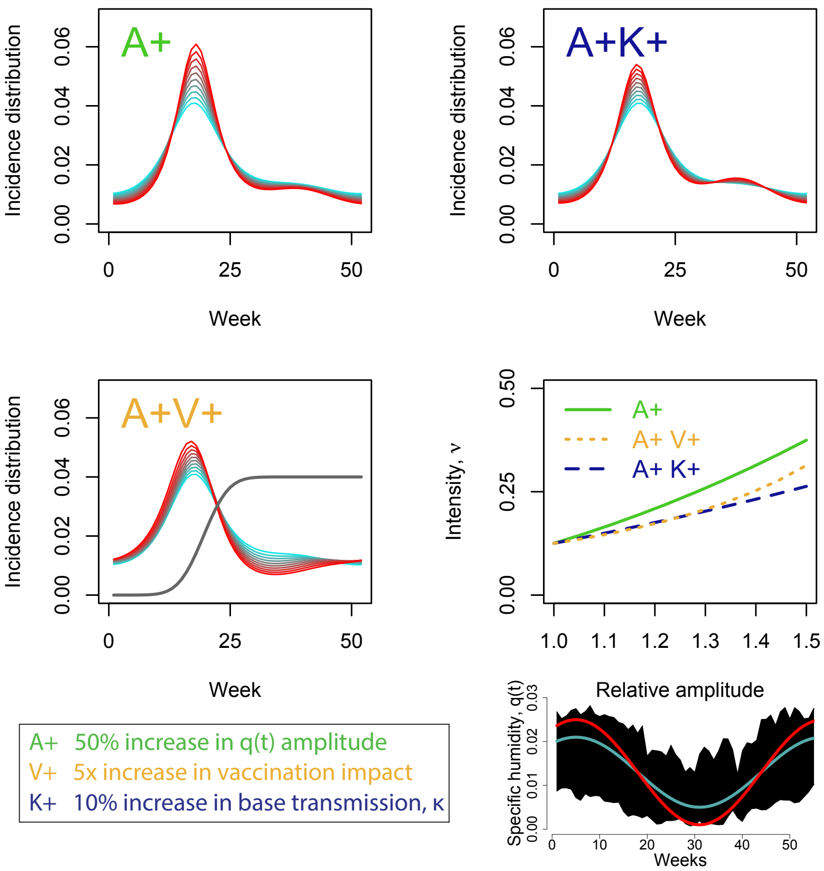

Figure S6. Modeling the impact of climate change, urbanization and vaccination on epidemic intensity. All parameters are the same as in Fig 2, unless otherwise stated. **(A)** Increasing the amplitude of specific humidity cycles by up to 1.5 times the 2002-2008 US mean (i.e. going from the blue curve to the red curve in the bottom right inset, wherein the black polygon encloses specific humidity data US cities from 2002-2008). Higher amplitude cycles correspond to warmer colors in the plot. **(B)** As in (A), but allowing base transmission potential to increase concurrently by up to a factor of 1.1. **(C)** As in (A) but modeling vaccination by changing the equation for susceptible dynamics to $dS/dt = -N^{-1}\beta SI + \delta(N - S - E - I) - dV/dt$. The rate of change in fraction vaccinated is given by $dV/dt = v \times exp(-0.02(t-25)^2)$ where time t is modulo 52 weeks and the nonspecific vaccination parameter v controls the overall impact of vaccination on the susceptible fraction. Vaccination moves individuals from the susceptible compartment to the removed compartment, where they are subject to the same immune waning conditions as if immunized by infection. The figure shows dynamics for the range $0<v<5$. The shape of $V(t)$ is shown in grey. **(D)** Associated changes in epidemic intensity for each experiment. Vaccination could mimic the population-level immunogenic consequences of increased base transmission potential, buffering against increases in epidemic intensity driven by increased amplitude in climate fluctuations. Considerable geographic and temporal uncertainty in climate predictions hinders precise assessment of resulting changes in flu dynamics, however increasing average temperature is expected to increase amplitude of SH cycles (*46*). Correspondingly, our simulations cover the full range from no change in amplitude, to extreme changes in amplitude associated with multidegree increases in mean temperature.

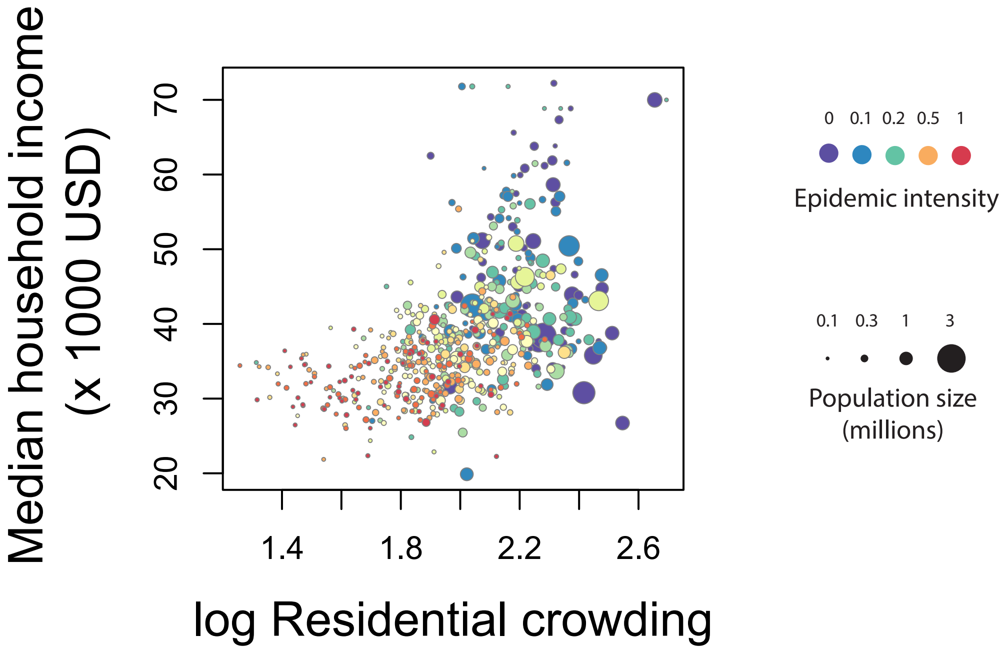

Figure S7. Correlations between epidemic intensity and socioeconomic conditions are enhanced by underlying variation in population size and crowding, which drive differences in base transmission potential. Median household income is higher, and epidemic intensity lower, in larger, more highly organized populations associated with urban centers. However, the majority of variation in intensity is associated with variation crowding and population size (moving horizontally on graph) rather than income per se (moving vertically on graph). Residential crowding is the same as shown in Figure 3E. Median household income is taken from US Census for the year 2000.

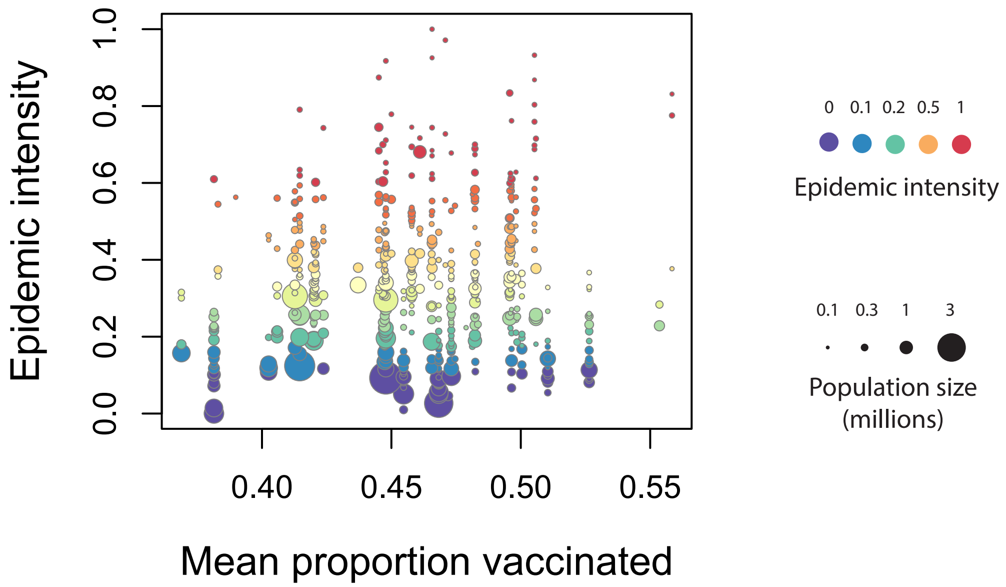

Figure S8. Variation among cities in epidemic intensity is not associated with state-level variation in vaccination coverage. Circle size is proportional to city population size, revealing the underlying association between epidemic intensity and population size. Reported mean proportion vaccinated is the mean for each state from 2010-2016, values for cities were interpolated from state-level data by assigning cities the value of their state.

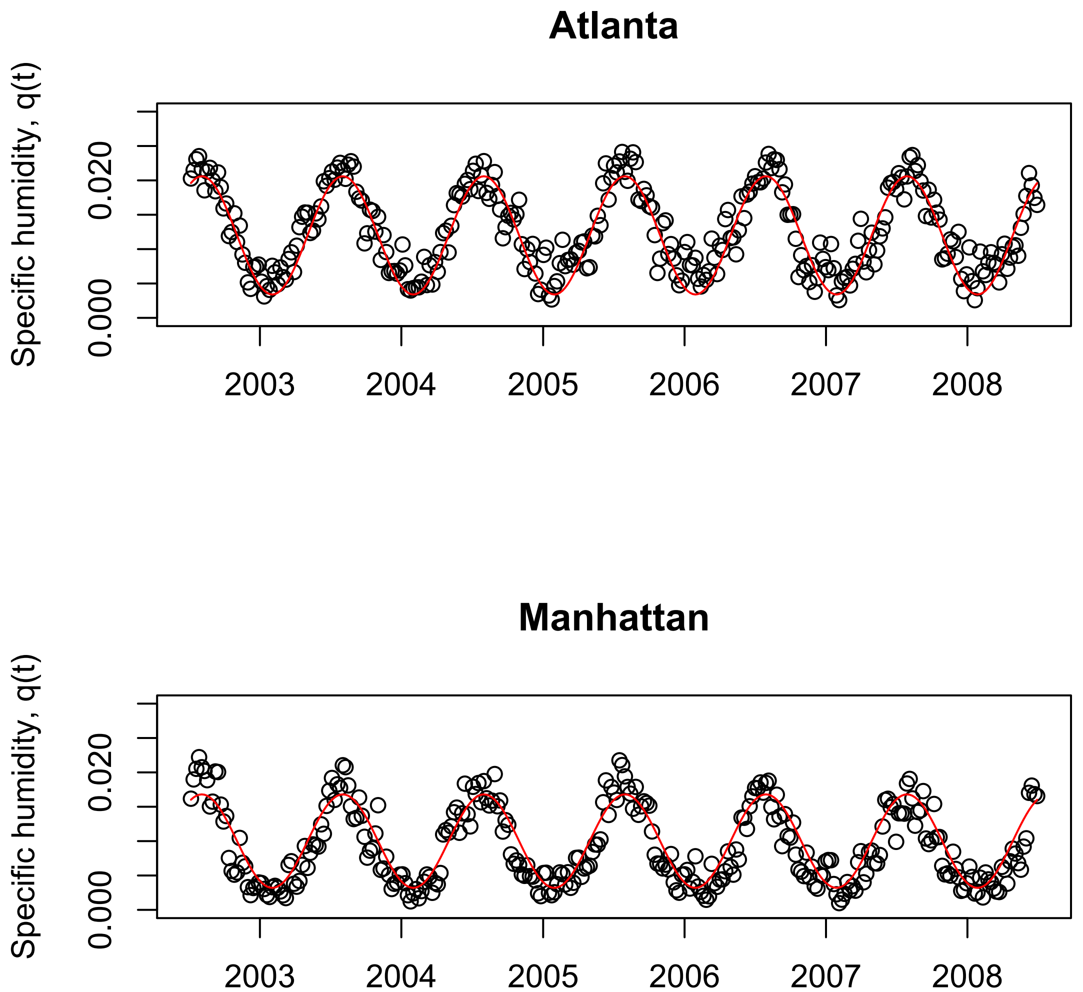

Figure S9. Specific humidity data (black circles) in Atlanta and Manhattan compared to the approximating function $q(t) = A \cos(2\pi (t - 5)/52) + B$ (red lines). The top panel uses A = 0.0086, B=0.012 and the bottom panel A = 0.0068, B = 0.010.

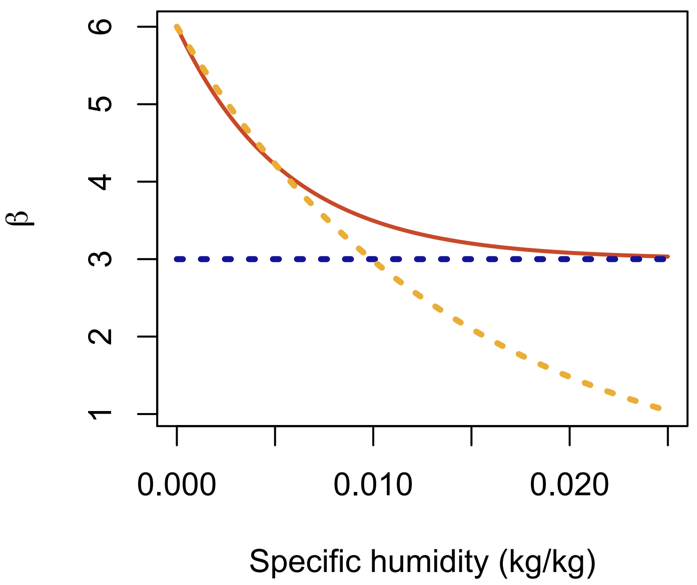

Figure S10. Approximating the transmission rate function. The original function $\beta(q) = \kappa + \sigma e^{-\omega q}$ *(red)*, is approximated with a piecewise function composed of $b_1(q) = (\kappa + \sigma)e^{-\omega 1 q}$ when q is small (yellow) and $b_2(q) = \kappa e^{-\omega 2 q}$ when q is large (blue). Parameter values: $\kappa = 3$, $\sigma = 3$, $\omega=180$, $\omega_1=70$, $\omega_2=0$.

Table S1. Summary statistics on the means of fitted model parameters across cities. Confidence intervals for base transmission rate κ in each city are shown in figure S5.

Parameter	Meaning	25th percentile	Median	Mean	75th percentile	Correlation with population size (Spearman's ρ)
κ	Base transmission potential	5.59	6.54	6.76	7.72	0.85
σ_1	Transmission gain 02/03	5.39	8.25	9.87	12.72	0.27
σ_2	Transmission gain 03/04	7.45	10.31	11.28	14.36	0.18
σ_3	Transmission gain 04/05	8.47	11.49	12.51	15.53	0.29
σ_4	Transmission gain 05/06	7.17	9.85	10.37	12.55	0.27
σ_5	Transmission gain 06/07	6.27	8.58	9.13	11.16	0.24
σ_6	Transmission gain 07/08	8.57	12.32	13.45	16.67	0.14
ρ_1	Off-peak reporting	0.06	0.19	0.22	0.38	-0.06
ρ_2	Peak reporting	3.12	4.36	5.02	6.28	-0.13
ω_1	Off-peak loss rate	4.13	6.14	6.77	8.93	0.08
ω_2	Peak loss rate	13.89	26.81	26.82	38.69	0.03

References and Notes

1. S. Gandon, T. Day, C. J. E. Metcalf, B. T. Grenfell, Forecasting Epidemiological and Evolutionary Dynamics of Infectious Diseases. *Trends Ecol. Evol.* **31**, 776–788 (2016). doi:10.1016/j.tree.2016.07.010 Medline

2. D. H. Morris, K. M. Gostic, S. Pompei, T. Bedford, M. Łuksza, R. A. Neher, B. T. Grenfell, M. Lässig, J. W. McCauley, Predictive Modeling of Influenza Shows the Promise of Applied Evolutionary Biology. *Trends Microbiol.* **26**, 102–118 (2018). doi:10.1016/j.tim.2017.09.004 Medline

3. K. Koelle, S. Cobey, B. Grenfell, M. Pascual, Epochal evolution shapes the phylodynamics of interpandemic influenza A (H3N2) in humans. *Science* **314**, 1898–1903 (2006). doi:10.1126/science.1132745 Medline

4. M. Łuksza, M. Lässig, A predictive fitness model for influenza. *Nature* **507**, 57–61 (2014). doi:10.1038/nature13087 Medline

5. D. Zinder, T. Bedford, E. B. Baskerville, R. J. Woods, M. Roy, M. Pascual, Seasonality in the migration and establishment of H3N2 Influenza lineages with epidemic growth and decline. *BMC Evol. Biol.* **14**, 272 (2014). doi:10.1186/s12862-014-0272-2 Medline

6. T. Bedford, S. Riley, I. G. Barr, S. Broor, M. Chadha, N. J. Cox, R. S. Daniels, C. P. Gunasekaran, A. C. Hurt, A. Kelso, A. Klimov, N. S. Lewis, X. Li, J. W. McCauley, T. Odagiri, V. Potdar, A. Rambaut, Y. Shu, E. Skepner, D. J. Smith, M. A. Suchard, M. Tashiro, D. Wang, X. Xu, P. Lemey, C. A. Russell, Global circulation patterns of seasonal influenza viruses vary with antigenic drift. *Nature* **523**, 217–220 (2015). doi:10.1038/nature14460 Medline

7. M. Lipsitch, C. Viboud, Influenza seasonality: Lifting the fog. *Proc. Natl. Acad. Sci. U.S.A.* **106**, 3645–3646 (2009). doi:10.1073/pnas.0900933106 Medline

8. S. Cauchemez, A. Bhattarai, T. L. Marchbanks, R. P. Fagan, S. Ostroff, N. M. Ferguson, D. Swerdlow, S. V. Sodha, M. E. Moll, F. J. Angulo, R. Palekar, W. R. Archer, L. Finelli; Pennsylvania H1N1 working group, Role of social networks in shaping disease transmission during a community outbreak of 2009 H1N1 pandemic influenza. *Proc. Natl. Acad. Sci. U.S.A.* **108**, 2825–2830 (2011). doi:10.1073/pnas.1008895108 Medline

9. L. Bourouiba, E. Dehandschoewercker, J. W. M. Bush, Violent expiratory events: On coughing and sneezing. *J. Fluid Mech.* **745**, 537–563 (2014). doi:10.1017/jfm.2014.88

10. C. Viboud, O. N. Bjørnstad, D. L. Smith, L. Simonsen, M. A. Miller, B. T. Grenfell, Synchrony, waves, and spatial hierarchies in the spread of influenza. *Science* **312**, 447–451 (2006). doi:10.1126/science.1125237 Medline

11. V. Charu, S. Zeger, J. Gog, O. N. Bjørnstad, S. Kissler, L. Simonsen, B. T. Grenfell, C. Viboud, Human mobility and the spatial transmission of influenza in the United States. *PLOS Comput. Biol.* **13**, e1005382 (2017). doi:10.1371/journal.pcbi.1005382 Medline

12. M. Tizzoni, P. Bajardi, C. Poletto, J. J. Ramasco, D. Balcan, B. Gonçalves, N. Perra, V. Colizza, A. Vespignani, Real-time numerical forecast of global epidemic spreading: Case study of 2009 A/H1N1pdm. *BMC Med.* **10**, 165 (2012). doi:10.1186/1741-7015-10-165 Medline

13. J. D. Tamerius, J. Shaman, W. J. Alonso, K. Bloom-Feshbach, C. K. Uejio, A. Comrie, C. Viboud, Environmental predictors of seasonal influenza epidemics across temperate and tropical climates. *PLOS Pathog.* **9**, e1003194 (2013). doi:10.1371/journal.ppat.1003194 Medline

14. J. Shaman, M. Kohn, Absolute humidity modulates influenza survival, transmission, and seasonality. *Proc. Natl. Acad. Sci. U.S.A.* **106**, 3243–3248 (2009). doi:10.1073/pnas.0806852106 Medline

15. T. Bedford, S. Cobey, P. Beerli, M. Pascual, Global migration dynamics underlie evolution and persistence of human influenza A (H3N2). *PLOS Pathog.* **6**, e1000918 (2010). doi:10.1371/journal.ppat.1000918 Medline

16. C. A. Russell, T. C. Jones, I. G. Barr, N. J. Cox, R. J. Garten, V. Gregory, I. D. Gust, A. W. Hampson, A. J. Hay, A. C. Hurt, J. C. de Jong, A. Kelso, A. I. Klimov, T. Kageyama, N. Komadina, A. S. Lapedes, Y. P. Lin, A. Mosterin, M. Obuchi, T. Odagiri, A. D. M. E. Osterhaus, G. F. Rimmelzwaan, M. W. Shaw, E. Skepner, K. Stohr, M. Tashiro, R. A. M. Fouchier, D. J. Smith, The global circulation of seasonal influenza A (H3N2) viruses. *Science* **320**, 340–346 (2008). doi:10.1126/science.1154137 Medline

17. F. Wen, T. Bedford, S. Cobey, Explaining the geographical origins of seasonal influenza A (H3N2). *Proc. Biol. Sci.* **283**, 20161312 (2016). doi:10.1098/rspb.2016.1312 Medline

18. S. Cauchemez, A.-J. Valleron, P.-Y. Boëlle, A. Flahault, N. M. Ferguson, Estimating the impact of school closure on influenza transmission from Sentinel data. *Nature* **452**, 750–754 (2008). doi:10.1038/nature06732 Medline

19. J. R. Gog, S. Ballesteros, C. Viboud, L. Simonsen, O. N. Bjornstad, J. Shaman, D. L. Chao, F. Khan, B. T. Grenfell, Spatial Transmission of 2009 Pandemic Influenza in the US. *PLOS Comput. Biol.* **10**, e1003635 (2014). doi:10.1371/journal.pcbi.1003635 Medline

20. I. Chattopadhyay, E. Kiciman, J. W. Elliott, J. L. Shaman, A. Rzhetsky, Conjunction of factors triggering waves of seasonal influenza. *eLife* **7**, e30756 (2018). doi:10.7554/eLife.30756 Medline

21. S. Pei, S. Kandula, W. Yang, J. Shaman, Forecasting the spatial transmission of influenza in the United States. *Proc. Natl. Acad. Sci. U.S.A.* **115**, 2752–2757 (2018). Medline

22. United Nations, Department of Economic and Social Affairs, Population Division, "World Urbanization Prospects" (2014), pp. 1–32.

23. J. B. Axelsen, R. Yaari, B. T. Grenfell, L. Stone, Multiannual forecasting of seasonal influenza dynamics reveals climatic and evolutionary drivers. *Proc. Natl. Acad. Sci. U.S.A.* **111**, 9538–9542 (2014). doi:10.1073/pnas.1321656111 Medline

24. W. Yang, M. Lipsitch, J. Shaman, Inference of seasonal and pandemic influenza transmission dynamics. *Proc. Natl. Acad. Sci. U.S.A.* **112**, 2723–2728 (2015). doi:10.1073/pnas.1415012112 Medline

25. R. J. Hatchett, C. E. Mecher, M. Lipsitch, Public health interventions and epidemic intensity during the 1918 influenza pandemic. *Proc. Natl. Acad. Sci. U.S.A.* **104**, 7582–7587 (2007). doi:10.1073/pnas.0610941104 Medline

26. E. M. Galarce, S. Minsky, K. Viswanath, Socioeconomic status, demographics, beliefs and A(H1N1) vaccine uptake in the United States. *Vaccine* **29**, 5284–5289 (2011). doi:10.1016/j.vaccine.2011.05.014 Medline

27. R. Chetty, M. Stepner, S. Abraham, S. Lin, B. Scuderi, N. Turner, A. Bergeron, D. Cutler, The Association Between Income and Life Expectancy in the United States, 2001-2014. *JAMA* **315**, 1750–1766 (2016). doi:10.1001/jama.2016.4226 Medline

28. K. H. Grantz, M. S. Rane, H. Salje, G. E. Glass, S. E. Schachterle, D. A. T. Cummings, Disparities in influenza mortality and transmission related to sociodemographic factors within Chicago in the pandemic of 1918. *Proc. Natl. Acad. Sci. U.S.A.* **113**, 13839–13844 (2016). doi:10.1073/pnas.1612838113 Medline

29. E. C. Lee, A. Arab, S. M. Goldlust, C. Viboud, B. T. Grenfell, S. Bansal, Deploying digital health data to optimize influenza surveillance at national and local scales. *PLOS Comput. Biol.* **14**, e1006020 (2018). doi:10.1371/journal.pcbi.1006020 Medline

30. L. M. A. Bettencourt, J. Lobo, D. Helbing, C. Kühnert, G. B. West, Growth, innovation, scaling, and the pace of life in cities. *Proc. Natl. Acad. Sci. U.S.A.* **104**, 7301–7306 (2007). doi:10.1073/pnas.0610172104 Medline

31. B. D. Dalziel, B. Pourbohloul, S. P. Ellner, Human mobility patterns predict divergent epidemic dynamics among cities. *Proc. Biol. Sci.* **280**, 20130763 (2013). doi:10.1098/rspb.2013.0763 Medline

32. B. D. Dalziel, K. Huang, J. L. Geoghegan, N. Arinaminpathy, E. J. Dubovi, B. T. Grenfell, S. P. Ellner, E. C. Holmes, C. R. Parrish, Contact heterogeneity, rather than transmission efficiency, limits the emergence and spread of canine influenza virus. *PLOS Pathog.* **10**, e1004455 (2014). doi:10.1371/journal.ppat.1004455 Medline

33. P. P. Martinez, A. A. King, M. Yunus, A. S. G. Faruque, M. Pascual, Differential and enhanced response to climate forcing in diarrheal disease due to rotavirus across a megacity of the developing world. *Proc. Natl. Acad. Sci. U.S.A.* **113**, 4092–4097 (2016). doi:10.1073/pnas.1518977113 Medline

34. C. Viboud, V. Charu, D. Olson, S. Ballesteros, J. Gog, F. Khan, B. Grenfell, L. Simonsen, Demonstrating the use of high-volume electronic medical claims data to monitor local and regional influenza activity in the US. *PLOS ONE* **9**, e102429 (2014). doi:10.1371/journal.pone.0102429 Medline

35. D. E. te Beest, M. van Boven, M. Hooiveld, C. van den Dool, J. Wallinga, Driving factors of influenza transmission in the Netherlands. *Am. J. Epidemiol.* **178**, 1469–1477 (2013). doi:10.1093/aje/kwt132 Medline

36. O. N. Bjørnstad, B. F. Finkenstadt, B. T. Grenfell, B. F. Finkenst a dt, B. T. Grenfell, Dynamics of measles epidemics: Estimating scaling of transmission rates using a time series SIR model. *Ecol. Monogr.* **72**, 169–184 (2002). doi:10.2307/3100023

37. B. D. Dalziel, O. N. Bjørnstad, W. G. van Panhuis, D. S. Burke, C. J. E. Metcalf, B. T. Grenfell, Persistent Chaos of Measles Epidemics in the Prevaccination United States Caused by a Small Change in Seasonal Transmission Patterns. *PLOS Comput. Biol.* **12**, e1004655 (2016). doi:10.1371/journal.pcbi.1004655 Medline

38. L. Held, S. Meyer, J. Bracher, Probabilistic forecasting in infectious disease epidemiology: The 13th Armitage lecture. *Stat. Med.* **36**, 3443–3460 (2017). doi:10.1002/sim.7363 Medline

39. M. Lloyd, Mean crowding. *J. Anim. Ecol.* **36**, 1–30 (1967). doi:10.2307/3012

40. N. Arinaminpathy, I. K. Kim, P. Gargiullo, M. Haber, I. M. Foppa, M. Gambhir, J. Bresee, Estimating Direct and Indirect Protective Effect of Influenza Vaccination in the United States. *Am. J. Epidemiol.* **186**, 92–100 (2017). doi:10.1093/aje/kwx037 Medline

41. J. M. Crawford, R. Stallone, F. Zhang, M. Gerolimatos, D. D. Korologos, C. Sweetapple, M. de Geronimo, Y. Dlugacz, D. M. Armellino, C. C. Ginocchio, Laboratory surge response to pandemic (H1N1) 2009 outbreak, New York City metropolitan area, USA. *Emerg. Infect. Dis.* **16**, 8–13 (2010). doi:10.3201/eid1601.091167 Medline

42. N. J. Hill, I. T. M. Hussein, K. R. Davis, E. J. Ma, T. J. Spivey, A. M. Ramey, W. B. Puryear, S. R. Das, R. A. Halpin, X. Lin, N. B. Fedorova, D. L. Suarez, W. M. Boyce, J. A. Runstadler, Reassortment of Influenza A Viruses in Wild Birds in Alaska before H5 Clade 2.3.4.4 Outbreaks. *Emerg. Infect. Dis.* **23**, 654–657 (2017). doi:10.3201/eid2304.161668 Medline

43. C. J. E. Metcalf, J. Farrar, F. T. Cutts, N. E. Basta, A. L. Graham, J. Lessler, N. M. Ferguson, D. S. Burke, B. T. Grenfell, Use of serological surveys to generate key insights into the changing global landscape of infectious disease. *Lancet* **388**, 728–730 (2016). doi:10.1016/S0140-6736(16)30164-7 Medline

44. K. S. Xue, T. Stevens-Ayers, A. P. Campbell, J. A. Englund, S. A. Pergam, M. Boeckh, J. D. Bloom, Parallel evolution of influenza across multiple spatiotemporal scales. *eLife* **6**, e26875 (2017). doi:10.7554/eLife.26875 Medline

45. G. J. Harper, Airborne micro-organisms: Survival tests with four viruses. *J. Hyg. (Lond.)* **59**, 479–486 (1961). doi:10.1017/S0022172400039176 Medline

46. E. M. Fischer, R. Knutti, Robust projections of combined humidity and temperature extremes. *Nat. Clim. Chang.* **3**, 126–130 (2013). doi:10.1038/nclimate1682
