# Supplementary figures and images for: Urbanization and humidity shape the intensity of influenza epidemics in U.S. cities (part 2 of 2)
Source: Science. 2018 Oct 5;362(6410):75–9. doi: 10.1126/science.aat6030 (PMC6510303; doi:10.1126/science.aat6030)

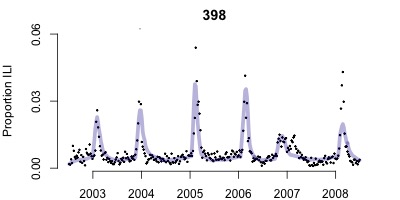

Supplement: Urbanization and humidity shape the intensity of influenza epidemics in U.S. cities [file Science-362-75-s002.zip › obs v sim comps all cities/398.jpg]

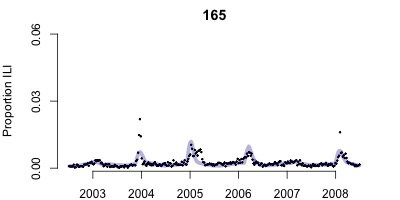

Supplement: Urbanization and humidity shape the intensity of influenza epidemics in U.S. cities [file Science-362-75-s002.zip › obs v sim comps all cities/165.jpg]

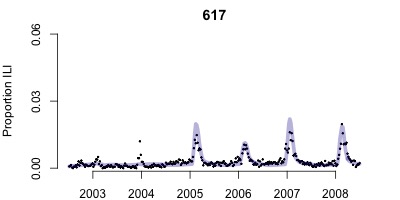

Supplement: Urbanization and humidity shape the intensity of influenza epidemics in U.S. cities [file Science-362-75-s002.zip › obs v sim comps all cities/617.jpg]

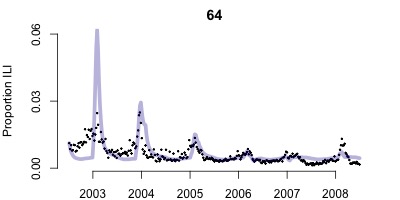

Supplement: Urbanization and humidity shape the intensity of influenza epidemics in U.S. cities [file Science-362-75-s002.zip › obs v sim comps all cities/64.jpg]

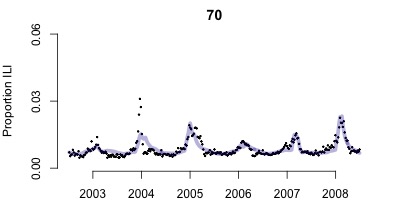

Supplement: Urbanization and humidity shape the intensity of influenza epidemics in U.S. cities [file Science-362-75-s002.zip › obs v sim comps all cities/70.jpg]

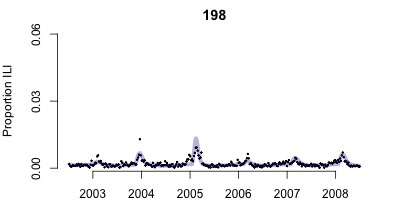

Supplement: Urbanization and humidity shape the intensity of influenza epidemics in U.S. cities [file Science-362-75-s002.zip › obs v sim comps all cities/198.jpg]

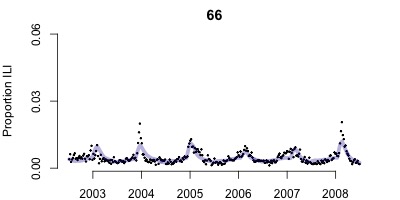

Supplement: Urbanization and humidity shape the intensity of influenza epidemics in U.S. cities [file Science-362-75-s002.zip › obs v sim comps all cities/66.jpg]

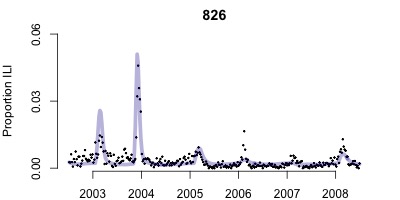

Supplement: Urbanization and humidity shape the intensity of influenza epidemics in U.S. cities [file Science-362-75-s002.zip › obs v sim comps all cities/826.jpg]

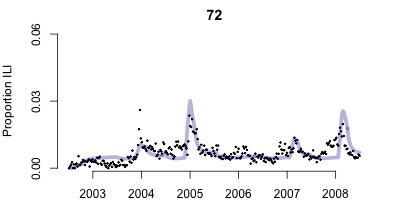

Supplement: Urbanization and humidity shape the intensity of influenza epidemics in U.S. cities [file Science-362-75-s002.zip › obs v sim comps all cities/72.jpg]

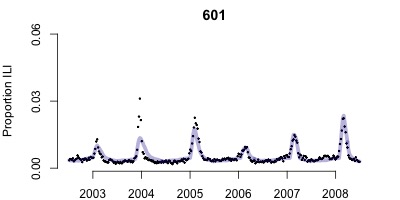

Supplement: Urbanization and humidity shape the intensity of influenza epidemics in U.S. cities [file Science-362-75-s002.zip › obs v sim comps all cities/601.jpg]

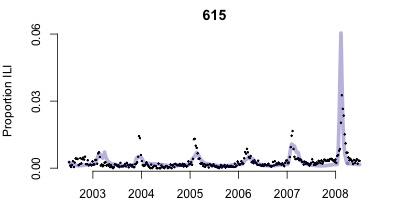

Supplement: Urbanization and humidity shape the intensity of influenza epidemics in U.S. cities [file Science-362-75-s002.zip › obs v sim comps all cities/615.jpg]

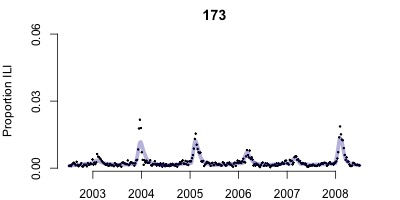

Supplement: Urbanization and humidity shape the intensity of influenza epidemics in U.S. cities [file Science-362-75-s002.zip › obs v sim comps all cities/173.jpg]

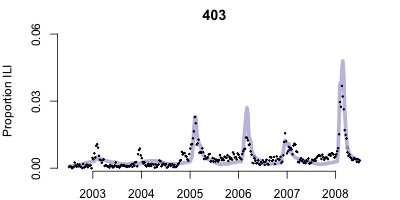

Supplement: Urbanization and humidity shape the intensity of influenza epidemics in U.S. cities [file Science-362-75-s002.zip › obs v sim comps all cities/403.jpg]

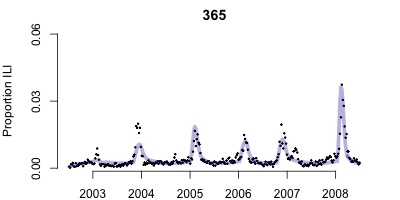

Supplement: Urbanization and humidity shape the intensity of influenza epidemics in U.S. cities [file Science-362-75-s002.zip › obs v sim comps all cities/365.jpg]

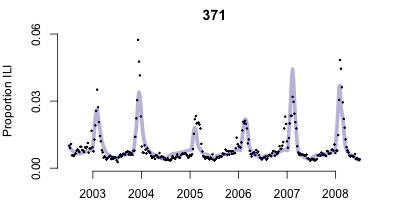

Supplement: Urbanization and humidity shape the intensity of influenza epidemics in U.S. cities [file Science-362-75-s002.zip › obs v sim comps all cities/371.jpg]

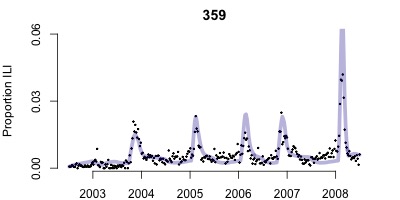

Supplement: Urbanization and humidity shape the intensity of influenza epidemics in U.S. cities [file Science-362-75-s002.zip › obs v sim comps all cities/359.jpg]

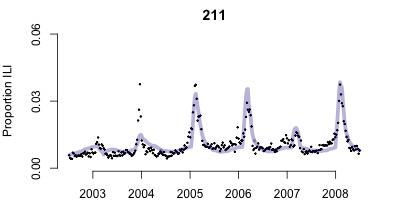

Supplement: Urbanization and humidity shape the intensity of influenza epidemics in U.S. cities [file Science-362-75-s002.zip › obs v sim comps all cities/211.jpg]

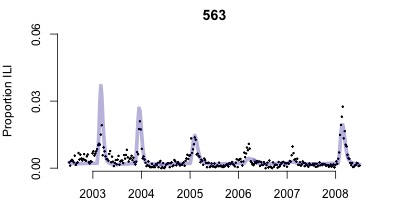

Supplement: Urbanization and humidity shape the intensity of influenza epidemics in U.S. cities [file Science-362-75-s002.zip › obs v sim comps all cities/563.jpg]

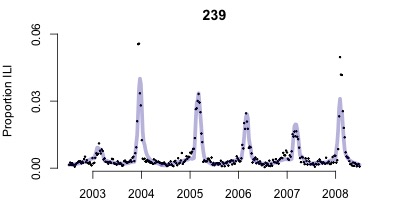

Supplement: Urbanization and humidity shape the intensity of influenza epidemics in U.S. cities [file Science-362-75-s002.zip › obs v sim comps all cities/239.jpg]

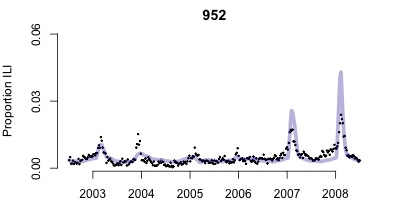

Supplement: Urbanization and humidity shape the intensity of influenza epidemics in U.S. cities [file Science-362-75-s002.zip › obs v sim comps all cities/952.jpg]

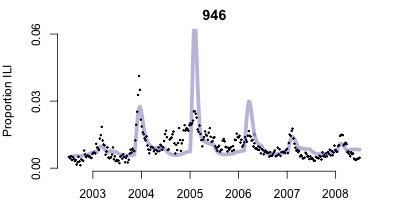

Supplement: Urbanization and humidity shape the intensity of influenza epidemics in U.S. cities [file Science-362-75-s002.zip › obs v sim comps all cities/946.jpg]

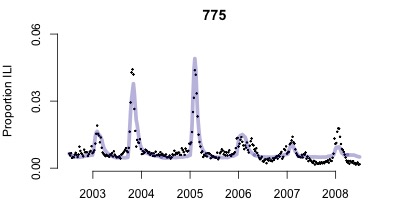

Supplement: Urbanization and humidity shape the intensity of influenza epidemics in U.S. cities [file Science-362-75-s002.zip › obs v sim comps all cities/775.jpg]

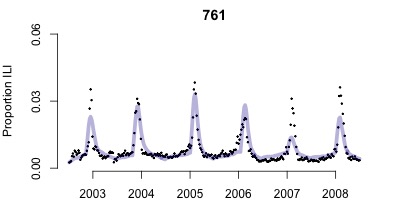

Supplement: Urbanization and humidity shape the intensity of influenza epidemics in U.S. cities [file Science-362-75-s002.zip › obs v sim comps all cities/761.jpg]

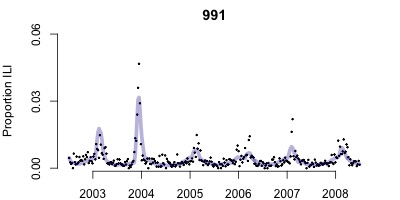

Supplement: Urbanization and humidity shape the intensity of influenza epidemics in U.S. cities [file Science-362-75-s002.zip › obs v sim comps all cities/991.jpg]

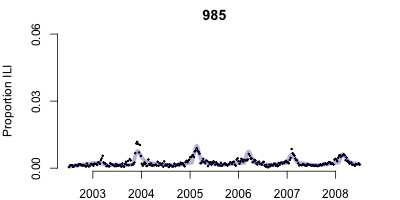

Supplement: Urbanization and humidity shape the intensity of influenza epidemics in U.S. cities [file Science-362-75-s002.zip › obs v sim comps all cities/985.jpg]

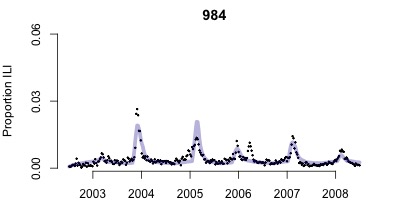

Supplement: Urbanization and humidity shape the intensity of influenza epidemics in U.S. cities [file Science-362-75-s002.zip › obs v sim comps all cities/984.jpg]

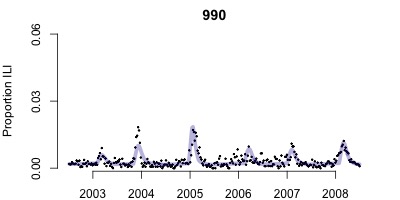

Supplement: Urbanization and humidity shape the intensity of influenza epidemics in U.S. cities [file Science-362-75-s002.zip › obs v sim comps all cities/990.jpg]

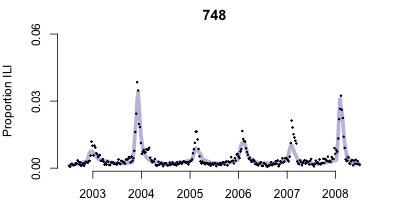

Supplement: Urbanization and humidity shape the intensity of influenza epidemics in U.S. cities [file Science-362-75-s002.zip › obs v sim comps all cities/748.jpg]

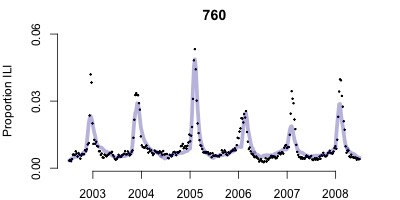

Supplement: Urbanization and humidity shape the intensity of influenza epidemics in U.S. cities [file Science-362-75-s002.zip › obs v sim comps all cities/760.jpg]

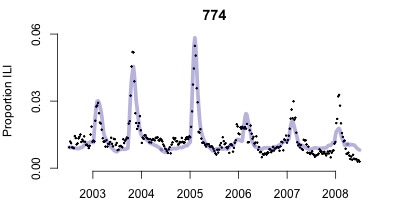

Supplement: Urbanization and humidity shape the intensity of influenza epidemics in U.S. cities [file Science-362-75-s002.zip › obs v sim comps all cities/774.jpg]

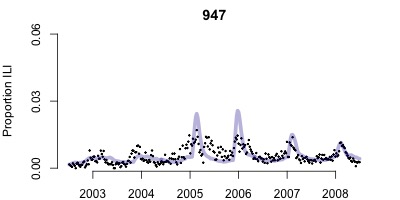

Supplement: Urbanization and humidity shape the intensity of influenza epidemics in U.S. cities [file Science-362-75-s002.zip › obs v sim comps all cities/947.jpg]

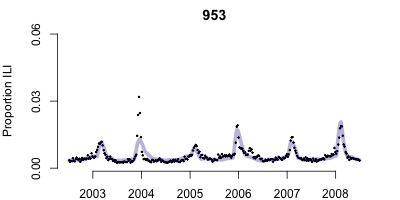

Supplement: Urbanization and humidity shape the intensity of influenza epidemics in U.S. cities [file Science-362-75-s002.zip › obs v sim comps all cities/953.jpg]

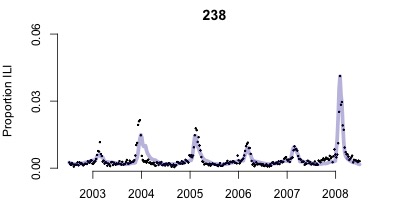

Supplement: Urbanization and humidity shape the intensity of influenza epidemics in U.S. cities [file Science-362-75-s002.zip › obs v sim comps all cities/238.jpg]

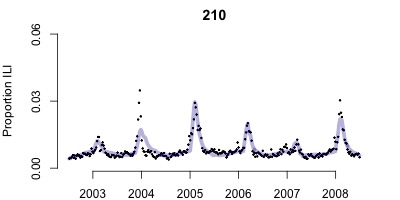

Supplement: Urbanization and humidity shape the intensity of influenza epidemics in U.S. cities [file Science-362-75-s002.zip › obs v sim comps all cities/210.jpg]

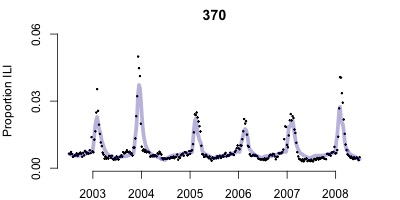

Supplement: Urbanization and humidity shape the intensity of influenza epidemics in U.S. cities [file Science-362-75-s002.zip › obs v sim comps all cities/370.jpg]

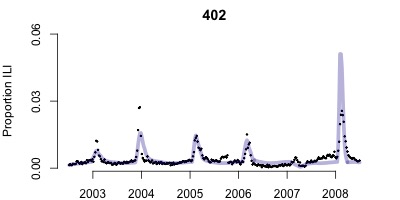

Supplement: Urbanization and humidity shape the intensity of influenza epidemics in U.S. cities [file Science-362-75-s002.zip › obs v sim comps all cities/402.jpg]

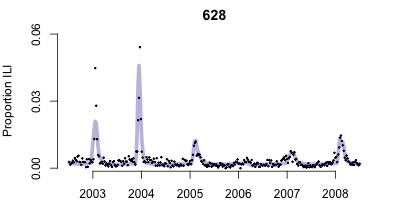

Supplement: Urbanization and humidity shape the intensity of influenza epidemics in U.S. cities [file Science-362-75-s002.zip › obs v sim comps all cities/628.jpg]

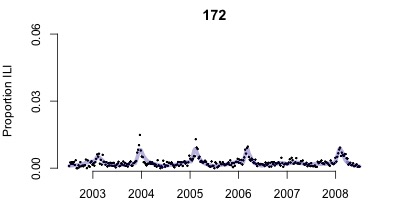

Supplement: Urbanization and humidity shape the intensity of influenza epidemics in U.S. cities [file Science-362-75-s002.zip › obs v sim comps all cities/172.jpg]

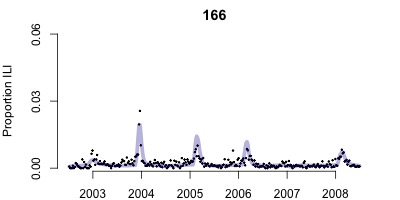

Supplement: Urbanization and humidity shape the intensity of influenza epidemics in U.S. cities [file Science-362-75-s002.zip › obs v sim comps all cities/166.jpg]

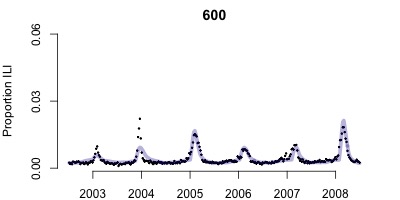

Supplement: Urbanization and humidity shape the intensity of influenza epidemics in U.S. cities [file Science-362-75-s002.zip › obs v sim comps all cities/600.jpg]

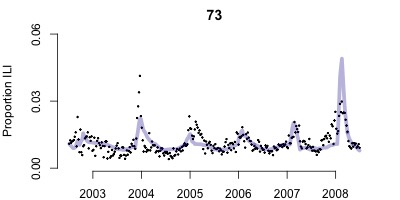

Supplement: Urbanization and humidity shape the intensity of influenza epidemics in U.S. cities [file Science-362-75-s002.zip › obs v sim comps all cities/73.jpg]

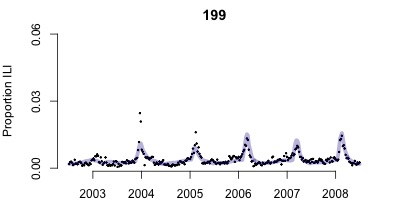

Supplement: Urbanization and humidity shape the intensity of influenza epidemics in U.S. cities [file Science-362-75-s002.zip › obs v sim comps all cities/199.jpg]

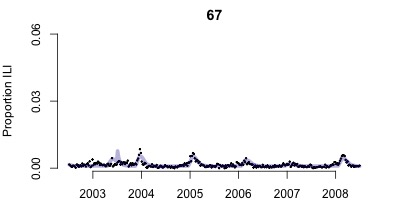

Supplement: Urbanization and humidity shape the intensity of influenza epidemics in U.S. cities [file Science-362-75-s002.zip › obs v sim comps all cities/67.jpg]

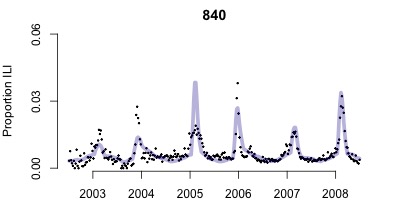

Supplement: Urbanization and humidity shape the intensity of influenza epidemics in U.S. cities [file Science-362-75-s002.zip › obs v sim comps all cities/840.jpg]

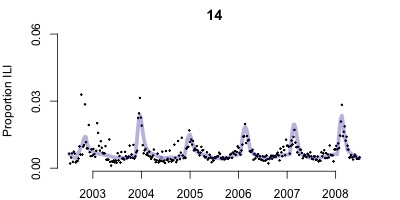

Supplement: Urbanization and humidity shape the intensity of influenza epidemics in U.S. cities [file Science-362-75-s002.zip › obs v sim comps all cities/14.jpg]

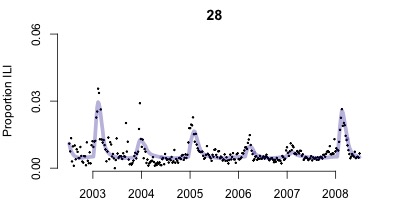

Supplement: Urbanization and humidity shape the intensity of influenza epidemics in U.S. cities [file Science-362-75-s002.zip › obs v sim comps all cities/28.jpg]

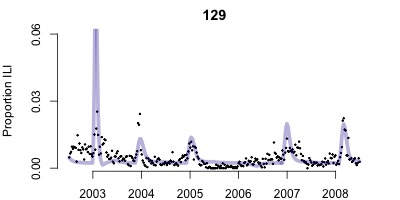

Supplement: Urbanization and humidity shape the intensity of influenza epidemics in U.S. cities [file Science-362-75-s002.zip › obs v sim comps all cities/129.jpg]

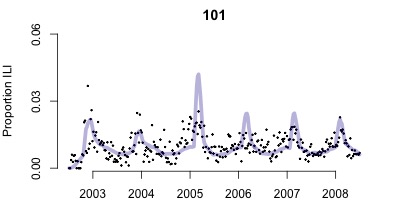

Supplement: Urbanization and humidity shape the intensity of influenza epidemics in U.S. cities [file Science-362-75-s002.zip › obs v sim comps all cities/101.jpg]

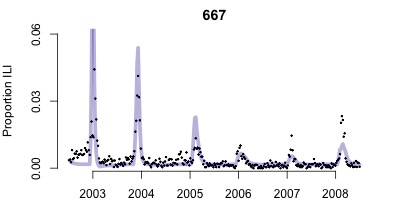

Supplement: Urbanization and humidity shape the intensity of influenza epidemics in U.S. cities [file Science-362-75-s002.zip › obs v sim comps all cities/667.jpg]

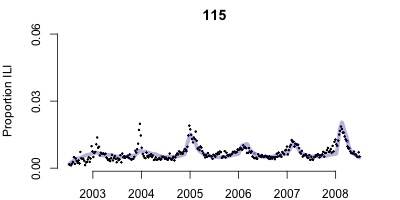

Supplement: Urbanization and humidity shape the intensity of influenza epidemics in U.S. cities [file Science-362-75-s002.zip › obs v sim comps all cities/115.jpg]

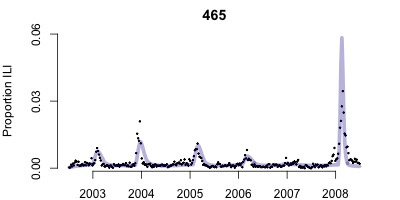

Supplement: Urbanization and humidity shape the intensity of influenza epidemics in U.S. cities [file Science-362-75-s002.zip › obs v sim comps all cities/465.jpg]

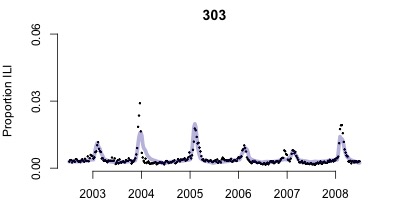

Supplement: Urbanization and humidity shape the intensity of influenza epidemics in U.S. cities [file Science-362-75-s002.zip › obs v sim comps all cities/303.jpg]

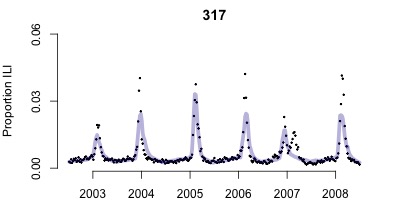

Supplement: Urbanization and humidity shape the intensity of influenza epidemics in U.S. cities [file Science-362-75-s002.zip › obs v sim comps all cities/317.jpg]

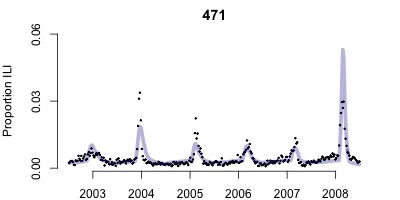

Supplement: Urbanization and humidity shape the intensity of influenza epidemics in U.S. cities [file Science-362-75-s002.zip › obs v sim comps all cities/471.jpg]

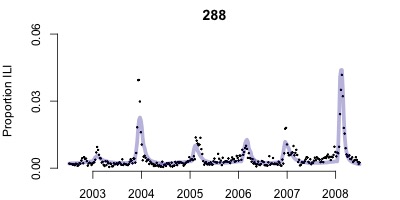

Supplement: Urbanization and humidity shape the intensity of influenza epidemics in U.S. cities [file Science-362-75-s002.zip › obs v sim comps all cities/288.jpg]

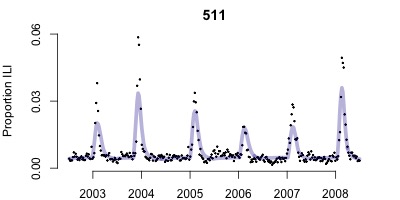

Supplement: Urbanization and humidity shape the intensity of influenza epidemics in U.S. cities [file Science-362-75-s002.zip › obs v sim comps all cities/511.jpg]

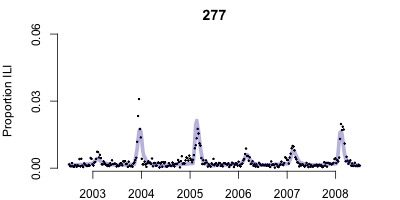

Supplement: Urbanization and humidity shape the intensity of influenza epidemics in U.S. cities [file Science-362-75-s002.zip › obs v sim comps all cities/277.jpg]

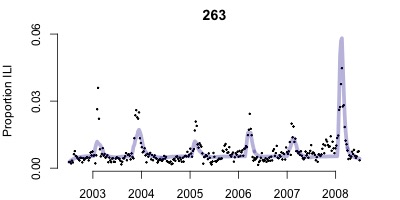

Supplement: Urbanization and humidity shape the intensity of influenza epidemics in U.S. cities [file Science-362-75-s002.zip › obs v sim comps all cities/263.jpg]

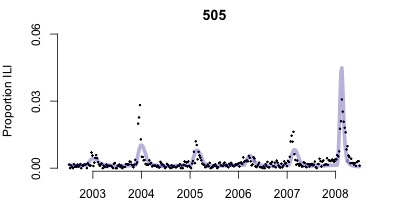

Supplement: Urbanization and humidity shape the intensity of influenza epidemics in U.S. cities [file Science-362-75-s002.zip › obs v sim comps all cities/505.jpg]

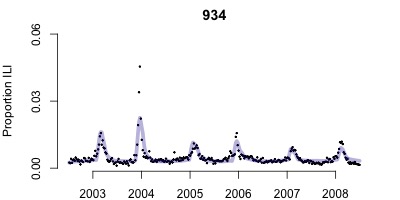

Supplement: Urbanization and humidity shape the intensity of influenza epidemics in U.S. cities [file Science-362-75-s002.zip › obs v sim comps all cities/934.jpg]

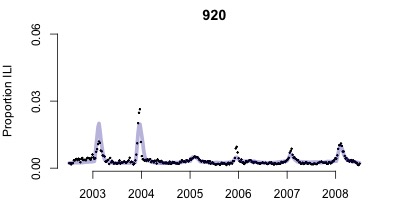

Supplement: Urbanization and humidity shape the intensity of influenza epidemics in U.S. cities [file Science-362-75-s002.zip › obs v sim comps all cities/920.jpg]

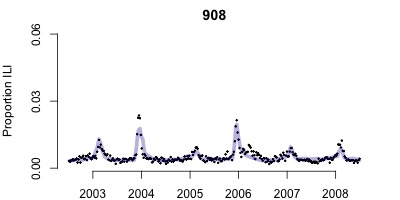

Supplement: Urbanization and humidity shape the intensity of influenza epidemics in U.S. cities [file Science-362-75-s002.zip › obs v sim comps all cities/908.jpg]

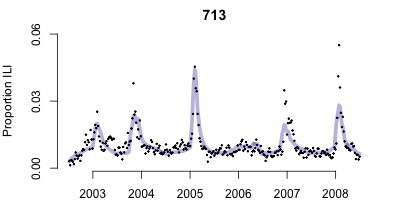

Supplement: Urbanization and humidity shape the intensity of influenza epidemics in U.S. cities [file Science-362-75-s002.zip › obs v sim comps all cities/713.jpg]

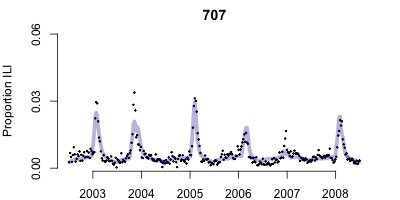

Supplement: Urbanization and humidity shape the intensity of influenza epidemics in U.S. cities [file Science-362-75-s002.zip › obs v sim comps all cities/707.jpg]

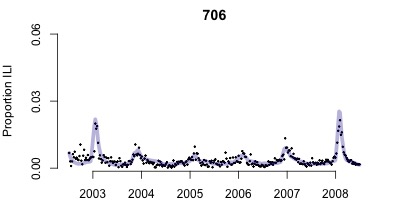

Supplement: Urbanization and humidity shape the intensity of influenza epidemics in U.S. cities [file Science-362-75-s002.zip › obs v sim comps all cities/706.jpg]

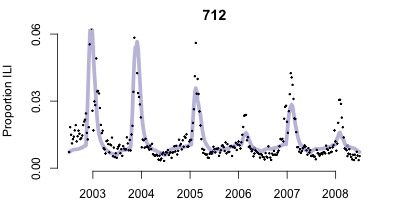

Supplement: Urbanization and humidity shape the intensity of influenza epidemics in U.S. cities [file Science-362-75-s002.zip › obs v sim comps all cities/712.jpg]

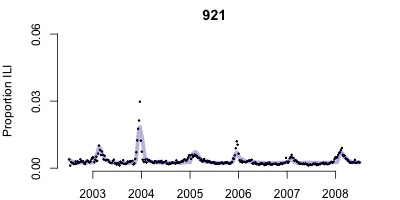

Supplement: Urbanization and humidity shape the intensity of influenza epidemics in U.S. cities [file Science-362-75-s002.zip › obs v sim comps all cities/921.jpg]

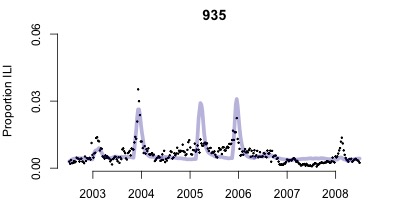

Supplement: Urbanization and humidity shape the intensity of influenza epidemics in U.S. cities [file Science-362-75-s002.zip › obs v sim comps all cities/935.jpg]

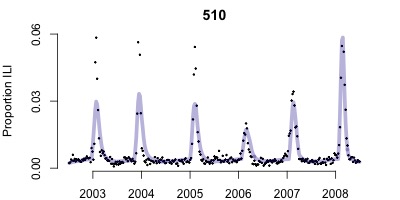

Supplement: Urbanization and humidity shape the intensity of influenza epidemics in U.S. cities [file Science-362-75-s002.zip › obs v sim comps all cities/510.jpg]

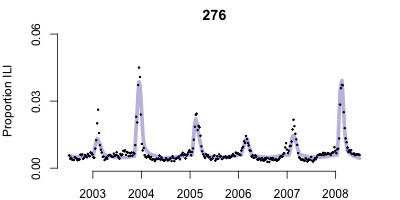

Supplement: Urbanization and humidity shape the intensity of influenza epidemics in U.S. cities [file Science-362-75-s002.zip › obs v sim comps all cities/276.jpg]

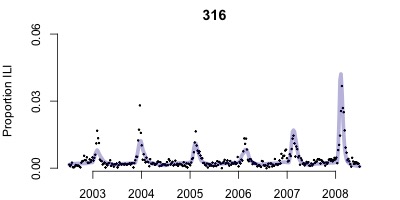

Supplement: Urbanization and humidity shape the intensity of influenza epidemics in U.S. cities [file Science-362-75-s002.zip › obs v sim comps all cities/316.jpg]

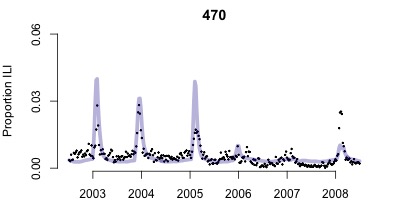

Supplement: Urbanization and humidity shape the intensity of influenza epidemics in U.S. cities [file Science-362-75-s002.zip › obs v sim comps all cities/470.jpg]

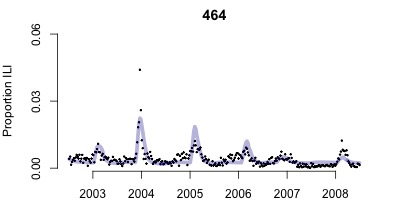

Supplement: Urbanization and humidity shape the intensity of influenza epidemics in U.S. cities [file Science-362-75-s002.zip › obs v sim comps all cities/464.jpg]

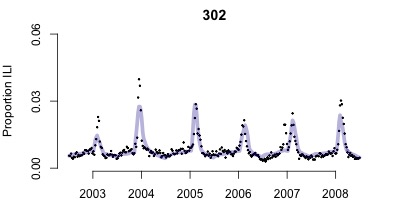

Supplement: Urbanization and humidity shape the intensity of influenza epidemics in U.S. cities [file Science-362-75-s002.zip › obs v sim comps all cities/302.jpg]

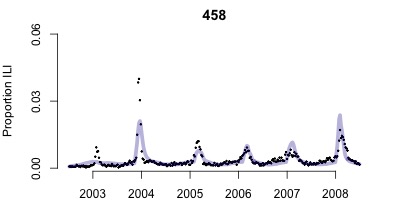

Supplement: Urbanization and humidity shape the intensity of influenza epidemics in U.S. cities [file Science-362-75-s002.zip › obs v sim comps all cities/458.jpg]

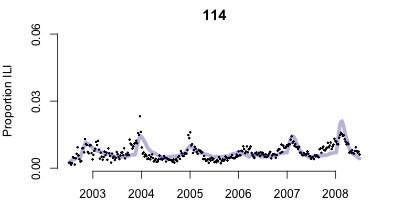

Supplement: Urbanization and humidity shape the intensity of influenza epidemics in U.S. cities [file Science-362-75-s002.zip › obs v sim comps all cities/114.jpg]

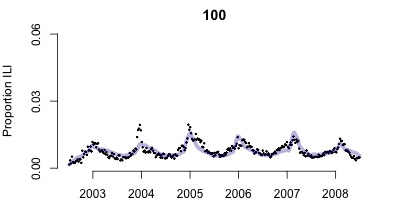

Supplement: Urbanization and humidity shape the intensity of influenza epidemics in U.S. cities [file Science-362-75-s002.zip › obs v sim comps all cities/100.jpg]

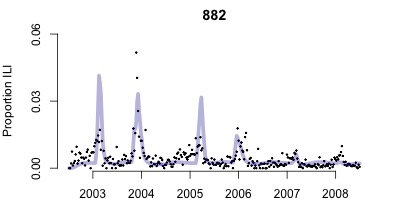

Supplement: Urbanization and humidity shape the intensity of influenza epidemics in U.S. cities [file Science-362-75-s002.zip › obs v sim comps all cities/882.jpg]

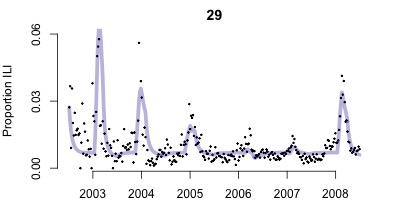

Supplement: Urbanization and humidity shape the intensity of influenza epidemics in U.S. cities [file Science-362-75-s002.zip › obs v sim comps all cities/29.jpg]

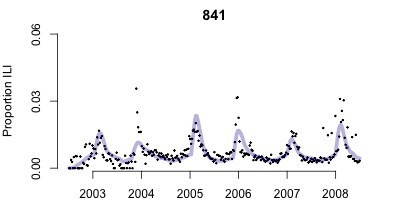

Supplement: Urbanization and humidity shape the intensity of influenza epidemics in U.S. cities [file Science-362-75-s002.zip › obs v sim comps all cities/841.jpg]

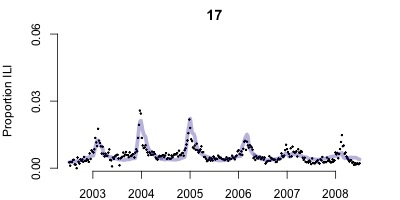

Supplement: Urbanization and humidity shape the intensity of influenza epidemics in U.S. cities [file Science-362-75-s002.zip › obs v sim comps all cities/17.jpg]

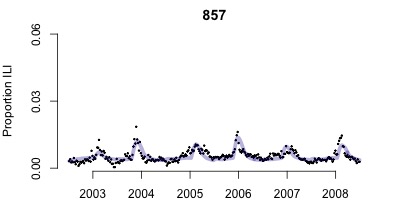

Supplement: Urbanization and humidity shape the intensity of influenza epidemics in U.S. cities [file Science-362-75-s002.zip › obs v sim comps all cities/857.jpg]

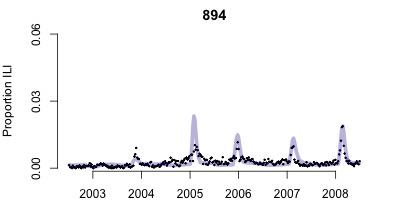

Supplement: Urbanization and humidity shape the intensity of influenza epidemics in U.S. cities [file Science-362-75-s002.zip › obs v sim comps all cities/894.jpg]

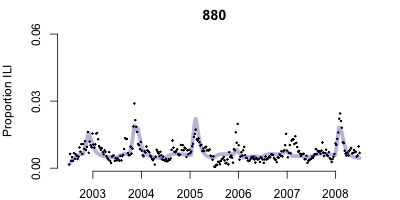

Supplement: Urbanization and humidity shape the intensity of influenza epidemics in U.S. cities [file Science-362-75-s002.zip › obs v sim comps all cities/880.jpg]

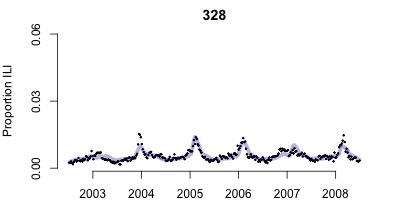

Supplement: Urbanization and humidity shape the intensity of influenza epidemics in U.S. cities [file Science-362-75-s002.zip › obs v sim comps all cities/328.jpg]

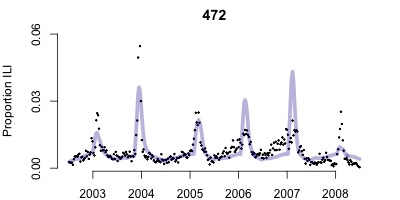

Supplement: Urbanization and humidity shape the intensity of influenza epidemics in U.S. cities [file Science-362-75-s002.zip › obs v sim comps all cities/472.jpg]

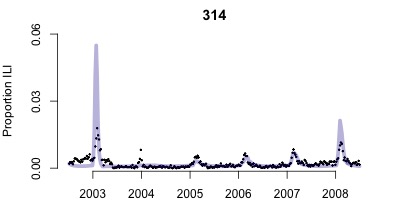

Supplement: Urbanization and humidity shape the intensity of influenza epidemics in U.S. cities [file Science-362-75-s002.zip › obs v sim comps all cities/314.jpg]

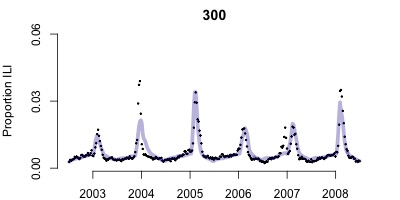

Supplement: Urbanization and humidity shape the intensity of influenza epidemics in U.S. cities [file Science-362-75-s002.zip › obs v sim comps all cities/300.jpg]

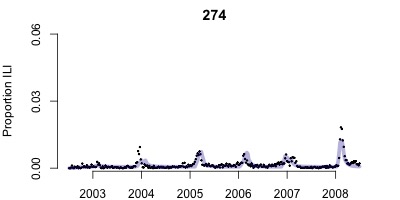

Supplement: Urbanization and humidity shape the intensity of influenza epidemics in U.S. cities [file Science-362-75-s002.zip › obs v sim comps all cities/274.jpg]

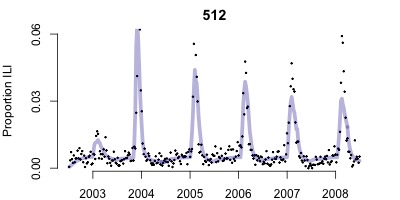

Supplement: Urbanization and humidity shape the intensity of influenza epidemics in U.S. cities [file Science-362-75-s002.zip › obs v sim comps all cities/512.jpg]

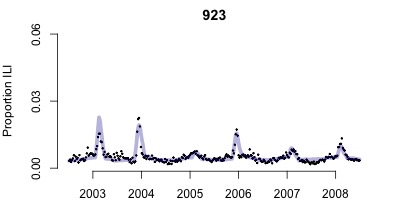

Supplement: Urbanization and humidity shape the intensity of influenza epidemics in U.S. cities [file Science-362-75-s002.zip › obs v sim comps all cities/923.jpg]

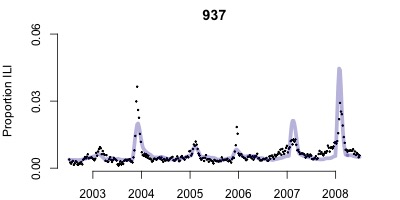

Supplement: Urbanization and humidity shape the intensity of influenza epidemics in U.S. cities [file Science-362-75-s002.zip › obs v sim comps all cities/937.jpg]

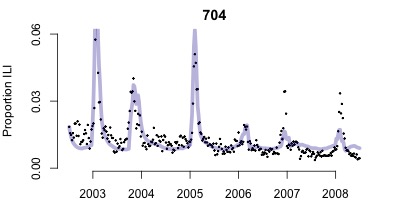

Supplement: Urbanization and humidity shape the intensity of influenza epidemics in U.S. cities [file Science-362-75-s002.zip › obs v sim comps all cities/704.jpg]

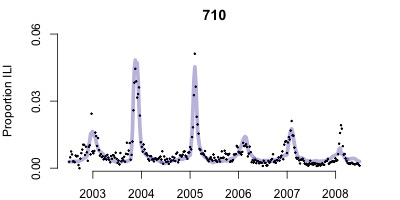

Supplement: Urbanization and humidity shape the intensity of influenza epidemics in U.S. cities [file Science-362-75-s002.zip › obs v sim comps all cities/710.jpg]

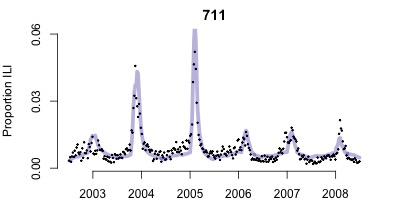

Supplement: Urbanization and humidity shape the intensity of influenza epidemics in U.S. cities [file Science-362-75-s002.zip › obs v sim comps all cities/711.jpg]

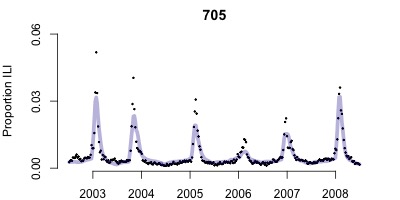

Supplement: Urbanization and humidity shape the intensity of influenza epidemics in U.S. cities [file Science-362-75-s002.zip › obs v sim comps all cities/705.jpg]

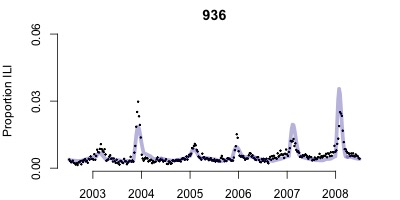

Supplement: Urbanization and humidity shape the intensity of influenza epidemics in U.S. cities [file Science-362-75-s002.zip › obs v sim comps all cities/936.jpg]

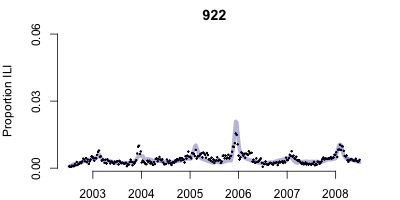

Supplement: Urbanization and humidity shape the intensity of influenza epidemics in U.S. cities [file Science-362-75-s002.zip › obs v sim comps all cities/922.jpg]

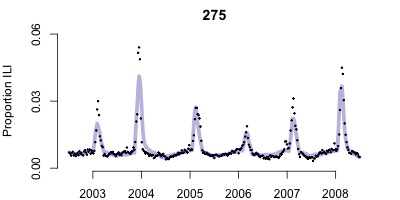

Supplement: Urbanization and humidity shape the intensity of influenza epidemics in U.S. cities [file Science-362-75-s002.zip › obs v sim comps all cities/275.jpg]

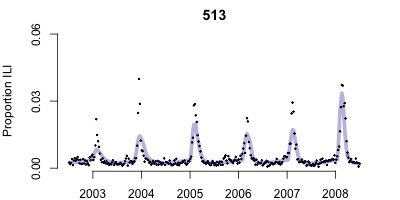

Supplement: Urbanization and humidity shape the intensity of influenza epidemics in U.S. cities [file Science-362-75-s002.zip › obs v sim comps all cities/513.jpg]
