# Supplementary figures and images for: Urbanization and humidity shape the intensity of influenza epidemics in U.S. cities (part 1 of 2)
Source: Science. 2018 Oct 5;362(6410):75–9. doi: 10.1126/science.aat6030 (PMC6510303; doi:10.1126/science.aat6030)

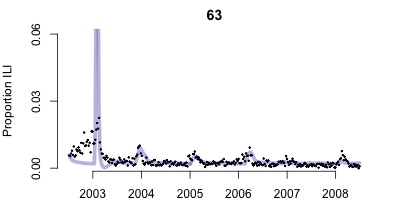

Supplement: Urbanization and humidity shape the intensity of influenza epidemics in U.S. cities [file Science-362-75-s002.zip › obs v sim comps all cities/63.jpg]

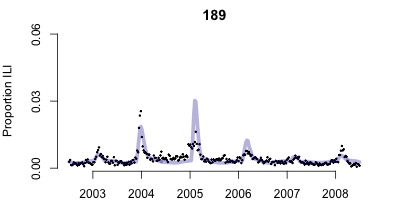

Supplement: Urbanization and humidity shape the intensity of influenza epidemics in U.S. cities [file Science-362-75-s002.zip › obs v sim comps all cities/189.jpg]

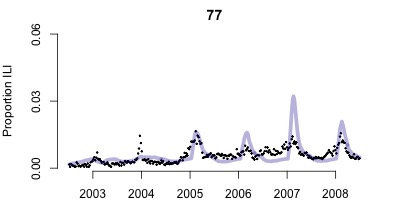

Supplement: Urbanization and humidity shape the intensity of influenza epidemics in U.S. cities [file Science-362-75-s002.zip › obs v sim comps all cities/77.jpg]

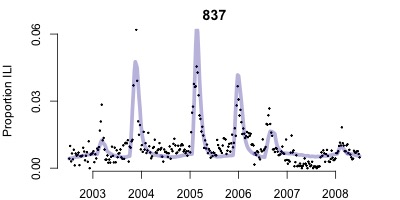

Supplement: Urbanization and humidity shape the intensity of influenza epidemics in U.S. cities [file Science-362-75-s002.zip › obs v sim comps all cities/837.jpg]

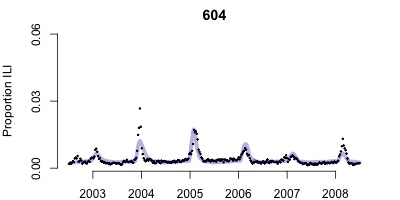

Supplement: Urbanization and humidity shape the intensity of influenza epidemics in U.S. cities [file Science-362-75-s002.zip › obs v sim comps all cities/604.jpg]

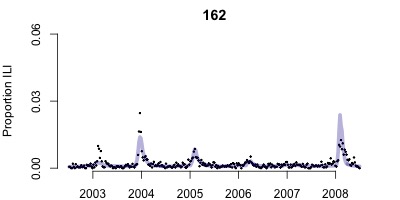

Supplement: Urbanization and humidity shape the intensity of influenza epidemics in U.S. cities [file Science-362-75-s002.zip › obs v sim comps all cities/162.jpg]

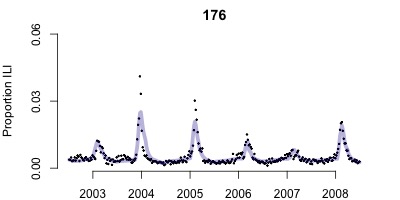

Supplement: Urbanization and humidity shape the intensity of influenza epidemics in U.S. cities [file Science-362-75-s002.zip › obs v sim comps all cities/176.jpg]

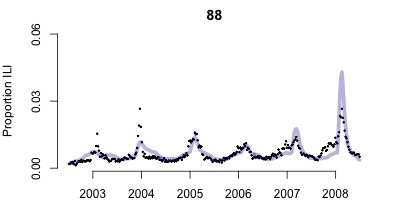

Supplement: Urbanization and humidity shape the intensity of influenza epidemics in U.S. cities [file Science-362-75-s002.zip › obs v sim comps all cities/88.jpg]

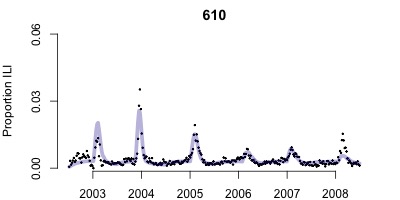

Supplement: Urbanization and humidity shape the intensity of influenza epidemics in U.S. cities [file Science-362-75-s002.zip › obs v sim comps all cities/610.jpg]

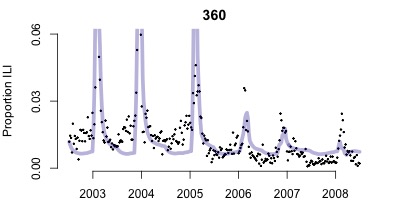

Supplement: Urbanization and humidity shape the intensity of influenza epidemics in U.S. cities [file Science-362-75-s002.zip › obs v sim comps all cities/360.jpg]

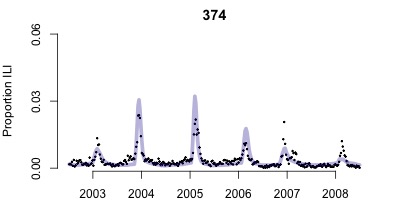

Supplement: Urbanization and humidity shape the intensity of influenza epidemics in U.S. cities [file Science-362-75-s002.zip › obs v sim comps all cities/374.jpg]

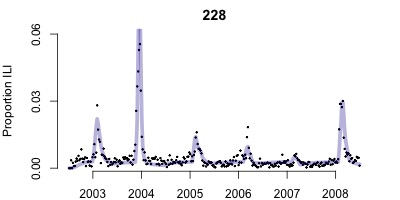

Supplement: Urbanization and humidity shape the intensity of influenza epidemics in U.S. cities [file Science-362-75-s002.zip › obs v sim comps all cities/228.jpg]

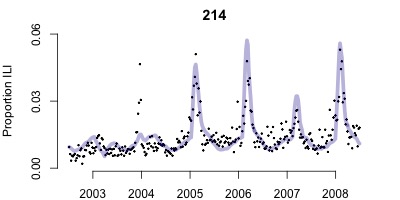

Supplement: Urbanization and humidity shape the intensity of influenza epidemics in U.S. cities [file Science-362-75-s002.zip › obs v sim comps all cities/214.jpg]

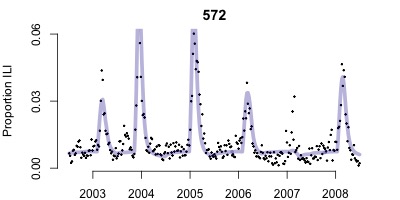

Supplement: Urbanization and humidity shape the intensity of influenza epidemics in U.S. cities [file Science-362-75-s002.zip › obs v sim comps all cities/572.jpg]

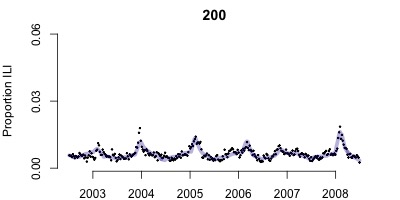

Supplement: Urbanization and humidity shape the intensity of influenza epidemics in U.S. cities [file Science-362-75-s002.zip › obs v sim comps all cities/200.jpg]

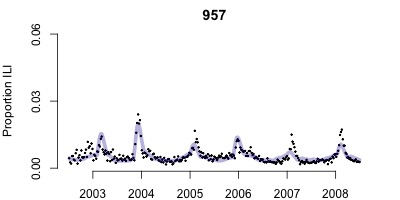

Supplement: Urbanization and humidity shape the intensity of influenza epidemics in U.S. cities [file Science-362-75-s002.zip › obs v sim comps all cities/957.jpg]

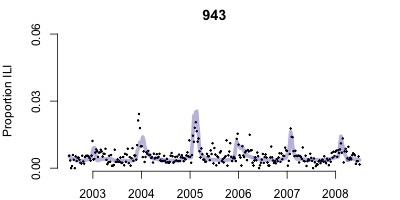

Supplement: Urbanization and humidity shape the intensity of influenza epidemics in U.S. cities [file Science-362-75-s002.zip › obs v sim comps all cities/943.jpg]

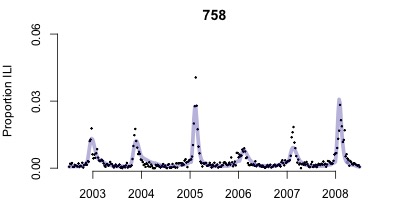

Supplement: Urbanization and humidity shape the intensity of influenza epidemics in U.S. cities [file Science-362-75-s002.zip › obs v sim comps all cities/758.jpg]

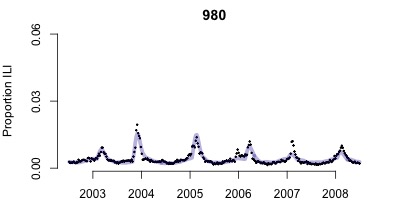

Supplement: Urbanization and humidity shape the intensity of influenza epidemics in U.S. cities [file Science-362-75-s002.zip › obs v sim comps all cities/980.jpg]

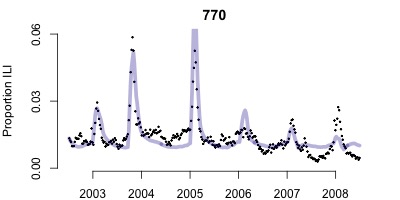

Supplement: Urbanization and humidity shape the intensity of influenza epidemics in U.S. cities [file Science-362-75-s002.zip › obs v sim comps all cities/770.jpg]

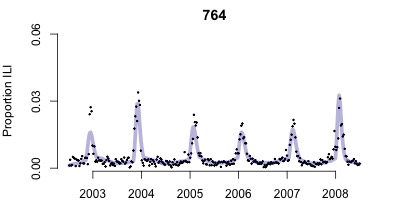

Supplement: Urbanization and humidity shape the intensity of influenza epidemics in U.S. cities [file Science-362-75-s002.zip › obs v sim comps all cities/764.jpg]

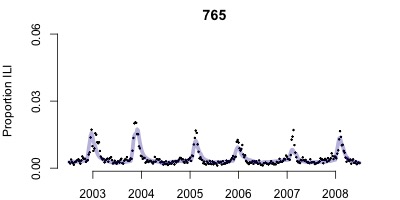

Supplement: Urbanization and humidity shape the intensity of influenza epidemics in U.S. cities [file Science-362-75-s002.zip › obs v sim comps all cities/765.jpg]

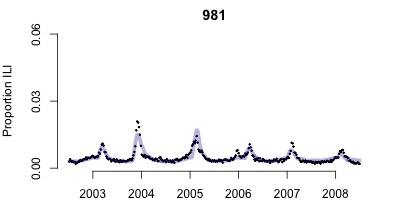

Supplement: Urbanization and humidity shape the intensity of influenza epidemics in U.S. cities [file Science-362-75-s002.zip › obs v sim comps all cities/981.jpg]

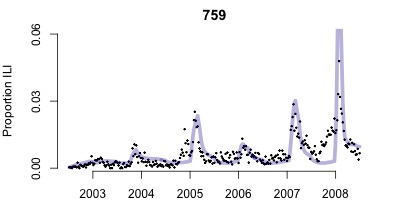

Supplement: Urbanization and humidity shape the intensity of influenza epidemics in U.S. cities [file Science-362-75-s002.zip › obs v sim comps all cities/759.jpg]

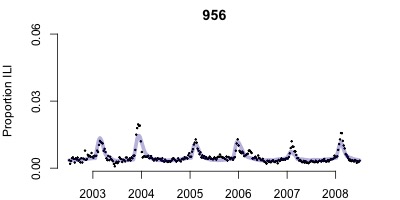

Supplement: Urbanization and humidity shape the intensity of influenza epidemics in U.S. cities [file Science-362-75-s002.zip › obs v sim comps all cities/956.jpg]

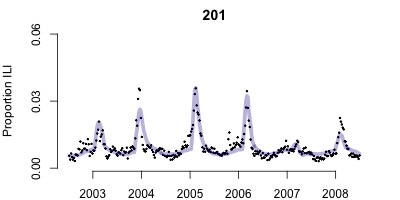

Supplement: Urbanization and humidity shape the intensity of influenza epidemics in U.S. cities [file Science-362-75-s002.zip › obs v sim comps all cities/201.jpg]

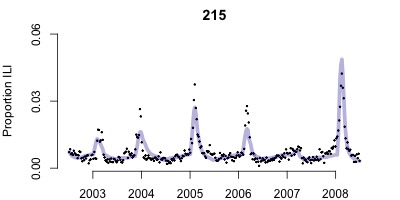

Supplement: Urbanization and humidity shape the intensity of influenza epidemics in U.S. cities [file Science-362-75-s002.zip › obs v sim comps all cities/215.jpg]

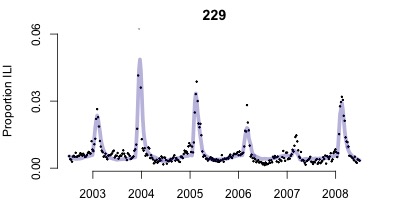

Supplement: Urbanization and humidity shape the intensity of influenza epidemics in U.S. cities [file Science-362-75-s002.zip › obs v sim comps all cities/229.jpg]

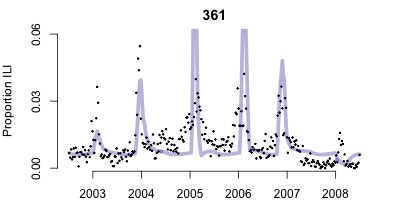

Supplement: Urbanization and humidity shape the intensity of influenza epidemics in U.S. cities [file Science-362-75-s002.zip › obs v sim comps all cities/361.jpg]

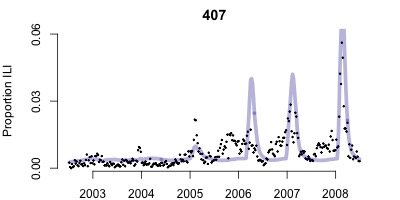

Supplement: Urbanization and humidity shape the intensity of influenza epidemics in U.S. cities [file Science-362-75-s002.zip › obs v sim comps all cities/407.jpg]

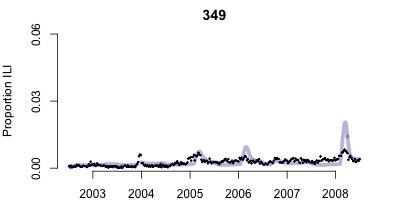

Supplement: Urbanization and humidity shape the intensity of influenza epidemics in U.S. cities [file Science-362-75-s002.zip › obs v sim comps all cities/349.jpg]

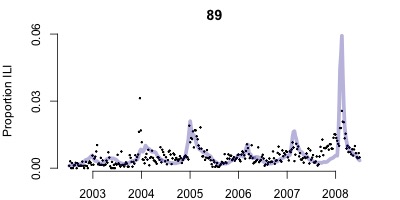

Supplement: Urbanization and humidity shape the intensity of influenza epidemics in U.S. cities [file Science-362-75-s002.zip › obs v sim comps all cities/89.jpg]

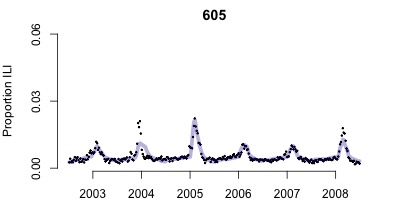

Supplement: Urbanization and humidity shape the intensity of influenza epidemics in U.S. cities [file Science-362-75-s002.zip › obs v sim comps all cities/605.jpg]

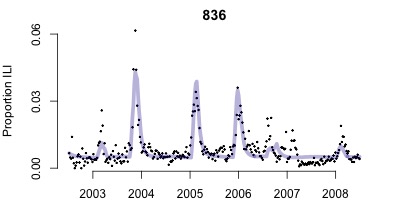

Supplement: Urbanization and humidity shape the intensity of influenza epidemics in U.S. cities [file Science-362-75-s002.zip › obs v sim comps all cities/836.jpg]

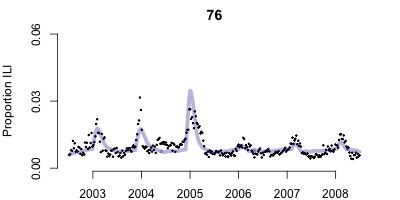

Supplement: Urbanization and humidity shape the intensity of influenza epidemics in U.S. cities [file Science-362-75-s002.zip › obs v sim comps all cities/76.jpg]

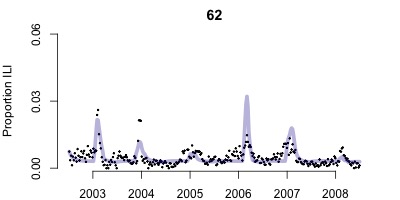

Supplement: Urbanization and humidity shape the intensity of influenza epidemics in U.S. cities [file Science-362-75-s002.zip › obs v sim comps all cities/62.jpg]

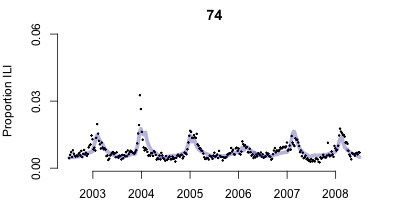

Supplement: Urbanization and humidity shape the intensity of influenza epidemics in U.S. cities [file Science-362-75-s002.zip › obs v sim comps all cities/74.jpg]

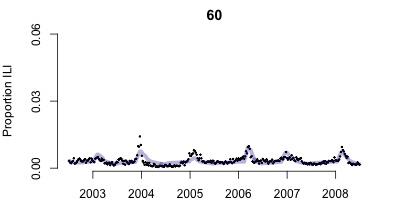

Supplement: Urbanization and humidity shape the intensity of influenza epidemics in U.S. cities [file Science-362-75-s002.zip › obs v sim comps all cities/60.jpg]

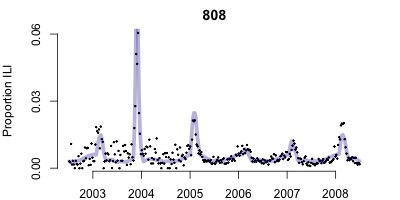

Supplement: Urbanization and humidity shape the intensity of influenza epidemics in U.S. cities [file Science-362-75-s002.zip › obs v sim comps all cities/808.jpg]

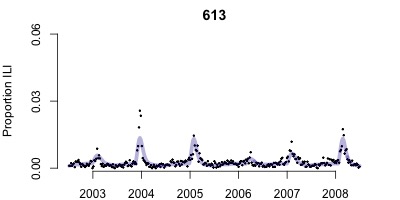

Supplement: Urbanization and humidity shape the intensity of influenza epidemics in U.S. cities [file Science-362-75-s002.zip › obs v sim comps all cities/613.jpg]

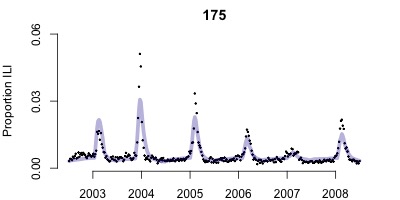

Supplement: Urbanization and humidity shape the intensity of influenza epidemics in U.S. cities [file Science-362-75-s002.zip › obs v sim comps all cities/175.jpg]

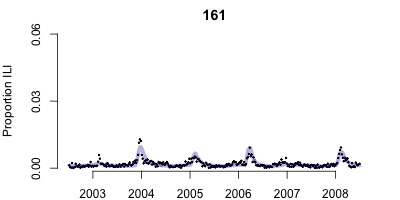

Supplement: Urbanization and humidity shape the intensity of influenza epidemics in U.S. cities [file Science-362-75-s002.zip › obs v sim comps all cities/161.jpg]

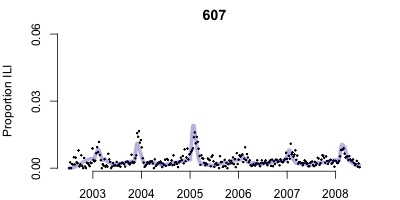

Supplement: Urbanization and humidity shape the intensity of influenza epidemics in U.S. cities [file Science-362-75-s002.zip › obs v sim comps all cities/607.jpg]

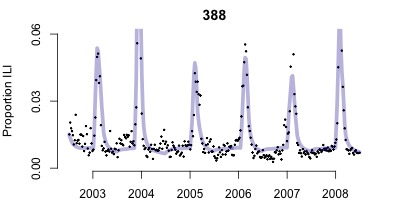

Supplement: Urbanization and humidity shape the intensity of influenza epidemics in U.S. cities [file Science-362-75-s002.zip › obs v sim comps all cities/388.jpg]

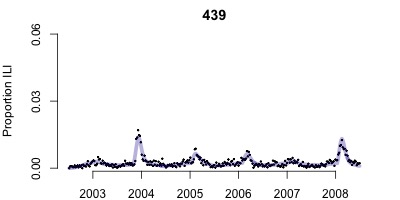

Supplement: Urbanization and humidity shape the intensity of influenza epidemics in U.S. cities [file Science-362-75-s002.zip › obs v sim comps all cities/439.jpg]

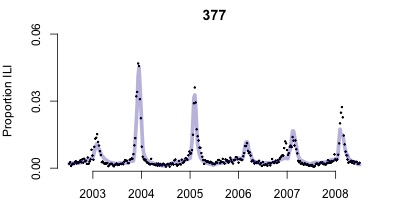

Supplement: Urbanization and humidity shape the intensity of influenza epidemics in U.S. cities [file Science-362-75-s002.zip › obs v sim comps all cities/377.jpg]

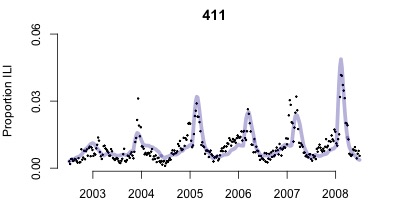

Supplement: Urbanization and humidity shape the intensity of influenza epidemics in U.S. cities [file Science-362-75-s002.zip › obs v sim comps all cities/411.jpg]

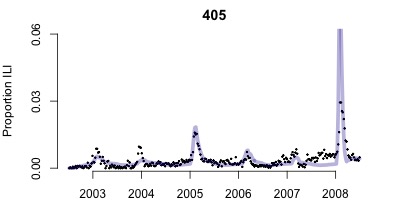

Supplement: Urbanization and humidity shape the intensity of influenza epidemics in U.S. cities [file Science-362-75-s002.zip › obs v sim comps all cities/405.jpg]

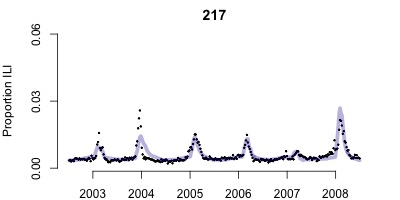

Supplement: Urbanization and humidity shape the intensity of influenza epidemics in U.S. cities [file Science-362-75-s002.zip › obs v sim comps all cities/217.jpg]

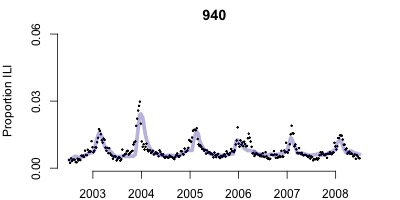

Supplement: Urbanization and humidity shape the intensity of influenza epidemics in U.S. cities [file Science-362-75-s002.zip › obs v sim comps all cities/940.jpg]

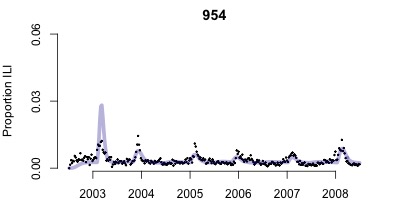

Supplement: Urbanization and humidity shape the intensity of influenza epidemics in U.S. cities [file Science-362-75-s002.zip › obs v sim comps all cities/954.jpg]

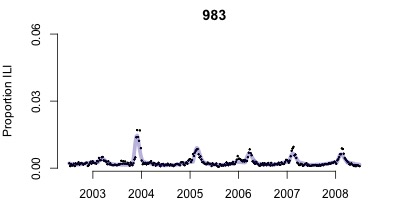

Supplement: Urbanization and humidity shape the intensity of influenza epidemics in U.S. cities [file Science-362-75-s002.zip › obs v sim comps all cities/983.jpg]

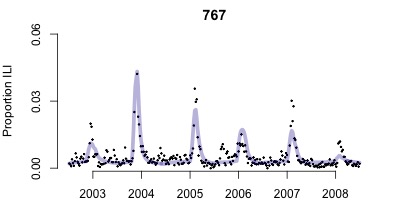

Supplement: Urbanization and humidity shape the intensity of influenza epidemics in U.S. cities [file Science-362-75-s002.zip › obs v sim comps all cities/767.jpg]

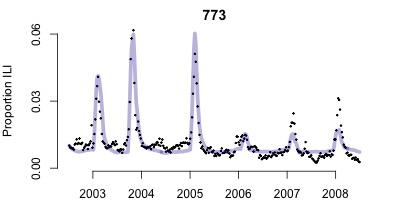

Supplement: Urbanization and humidity shape the intensity of influenza epidemics in U.S. cities [file Science-362-75-s002.zip › obs v sim comps all cities/773.jpg]

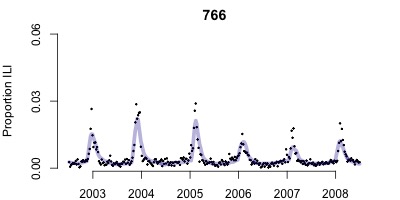

Supplement: Urbanization and humidity shape the intensity of influenza epidemics in U.S. cities [file Science-362-75-s002.zip › obs v sim comps all cities/766.jpg]

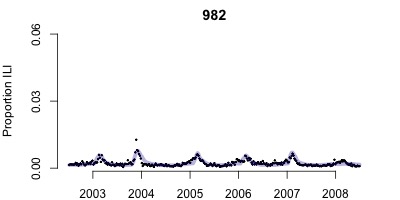

Supplement: Urbanization and humidity shape the intensity of influenza epidemics in U.S. cities [file Science-362-75-s002.zip › obs v sim comps all cities/982.jpg]

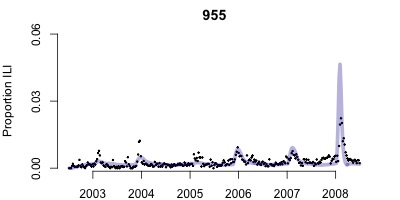

Supplement: Urbanization and humidity shape the intensity of influenza epidemics in U.S. cities [file Science-362-75-s002.zip › obs v sim comps all cities/955.jpg]

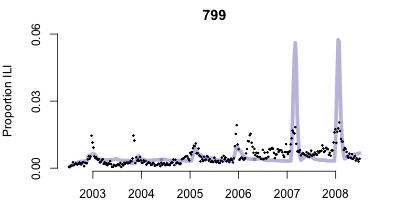

Supplement: Urbanization and humidity shape the intensity of influenza epidemics in U.S. cities [file Science-362-75-s002.zip › obs v sim comps all cities/799.jpg]

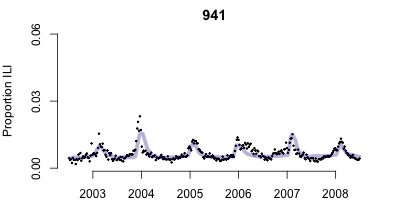

Supplement: Urbanization and humidity shape the intensity of influenza epidemics in U.S. cities [file Science-362-75-s002.zip › obs v sim comps all cities/941.jpg]

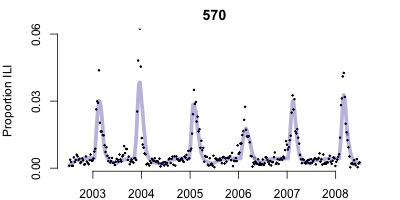

Supplement: Urbanization and humidity shape the intensity of influenza epidemics in U.S. cities [file Science-362-75-s002.zip › obs v sim comps all cities/570.jpg]

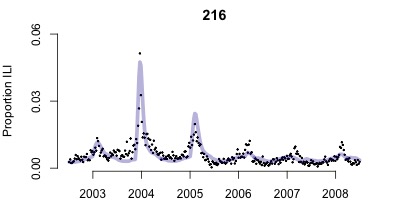

Supplement: Urbanization and humidity shape the intensity of influenza epidemics in U.S. cities [file Science-362-75-s002.zip › obs v sim comps all cities/216.jpg]

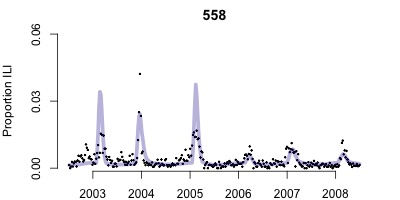

Supplement: Urbanization and humidity shape the intensity of influenza epidemics in U.S. cities [file Science-362-75-s002.zip › obs v sim comps all cities/558.jpg]

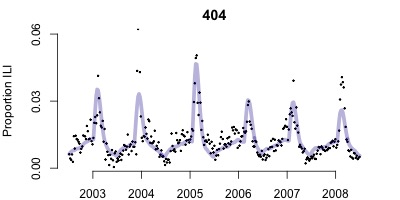

Supplement: Urbanization and humidity shape the intensity of influenza epidemics in U.S. cities [file Science-362-75-s002.zip › obs v sim comps all cities/404.jpg]

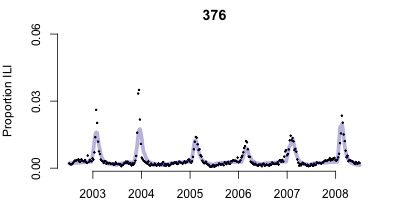

Supplement: Urbanization and humidity shape the intensity of influenza epidemics in U.S. cities [file Science-362-75-s002.zip › obs v sim comps all cities/376.jpg]

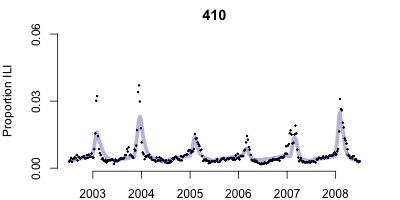

Supplement: Urbanization and humidity shape the intensity of influenza epidemics in U.S. cities [file Science-362-75-s002.zip › obs v sim comps all cities/410.jpg]

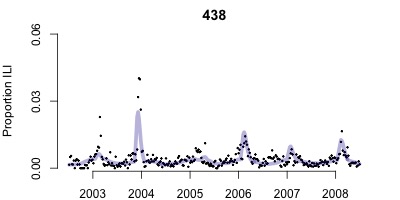

Supplement: Urbanization and humidity shape the intensity of influenza epidemics in U.S. cities [file Science-362-75-s002.zip › obs v sim comps all cities/438.jpg]

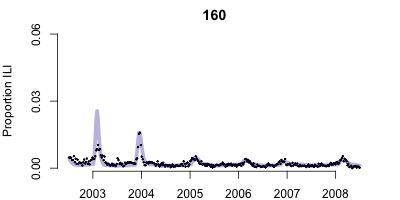

Supplement: Urbanization and humidity shape the intensity of influenza epidemics in U.S. cities [file Science-362-75-s002.zip › obs v sim comps all cities/160.jpg]

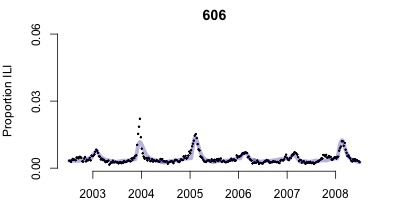

Supplement: Urbanization and humidity shape the intensity of influenza epidemics in U.S. cities [file Science-362-75-s002.zip › obs v sim comps all cities/606.jpg]

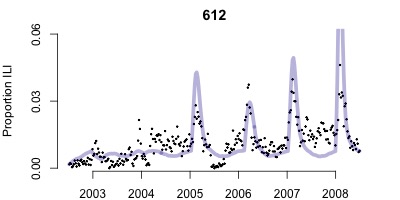

Supplement: Urbanization and humidity shape the intensity of influenza epidemics in U.S. cities [file Science-362-75-s002.zip › obs v sim comps all cities/612.jpg]

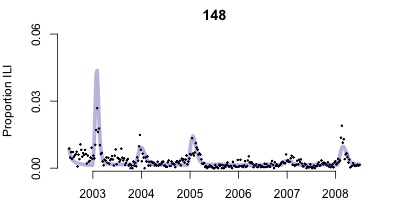

Supplement: Urbanization and humidity shape the intensity of influenza epidemics in U.S. cities [file Science-362-75-s002.zip › obs v sim comps all cities/148.jpg]

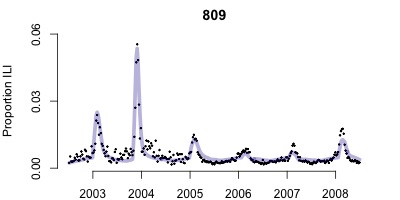

Supplement: Urbanization and humidity shape the intensity of influenza epidemics in U.S. cities [file Science-362-75-s002.zip › obs v sim comps all cities/809.jpg]

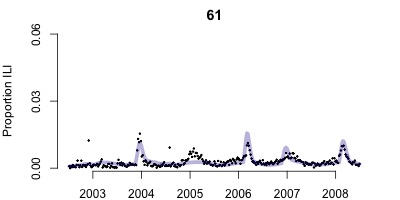

Supplement: Urbanization and humidity shape the intensity of influenza epidemics in U.S. cities [file Science-362-75-s002.zip › obs v sim comps all cities/61.jpg]

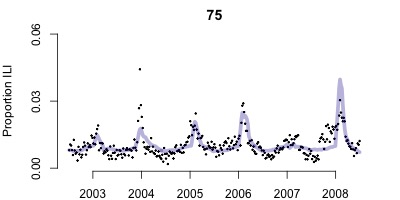

Supplement: Urbanization and humidity shape the intensity of influenza epidemics in U.S. cities [file Science-362-75-s002.zip › obs v sim comps all cities/75.jpg]

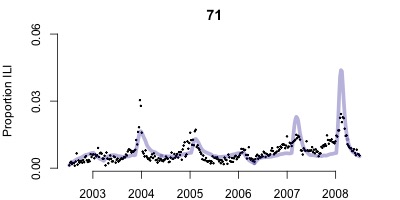

Supplement: Urbanization and humidity shape the intensity of influenza epidemics in U.S. cities [file Science-362-75-s002.zip › obs v sim comps all cities/71.jpg]

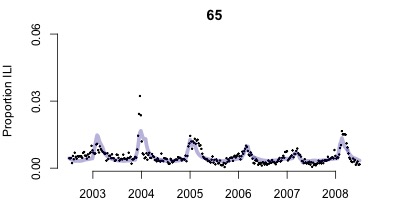

Supplement: Urbanization and humidity shape the intensity of influenza epidemics in U.S. cities [file Science-362-75-s002.zip › obs v sim comps all cities/65.jpg]

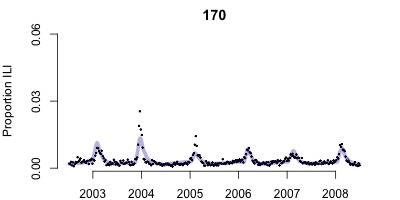

Supplement: Urbanization and humidity shape the intensity of influenza epidemics in U.S. cities [file Science-362-75-s002.zip › obs v sim comps all cities/170.jpg]

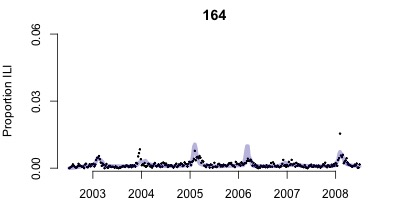

Supplement: Urbanization and humidity shape the intensity of influenza epidemics in U.S. cities [file Science-362-75-s002.zip › obs v sim comps all cities/164.jpg]

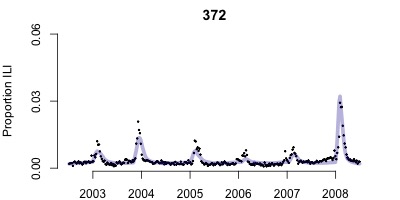

Supplement: Urbanization and humidity shape the intensity of influenza epidemics in U.S. cities [file Science-362-75-s002.zip › obs v sim comps all cities/372.jpg]

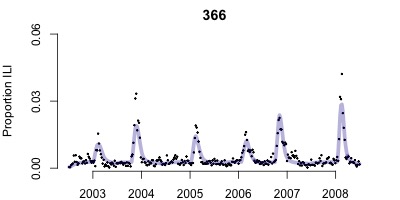

Supplement: Urbanization and humidity shape the intensity of influenza epidemics in U.S. cities [file Science-362-75-s002.zip › obs v sim comps all cities/366.jpg]

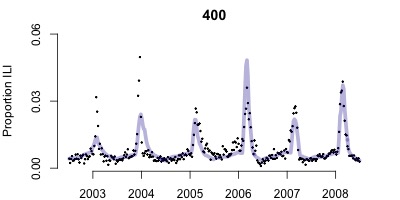

Supplement: Urbanization and humidity shape the intensity of influenza epidemics in U.S. cities [file Science-362-75-s002.zip › obs v sim comps all cities/400.jpg]

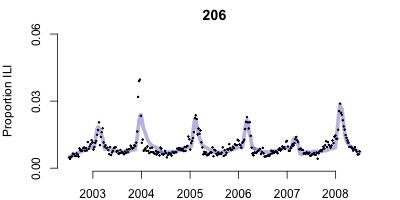

Supplement: Urbanization and humidity shape the intensity of influenza epidemics in U.S. cities [file Science-362-75-s002.zip › obs v sim comps all cities/206.jpg]

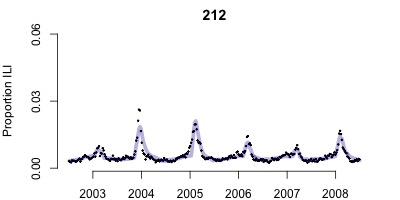

Supplement: Urbanization and humidity shape the intensity of influenza epidemics in U.S. cities [file Science-362-75-s002.zip › obs v sim comps all cities/212.jpg]

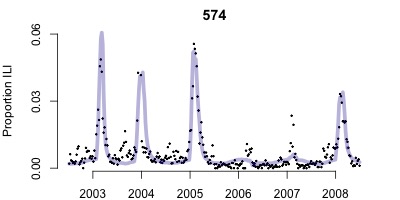

Supplement: Urbanization and humidity shape the intensity of influenza epidemics in U.S. cities [file Science-362-75-s002.zip › obs v sim comps all cities/574.jpg]

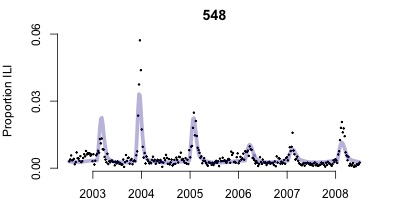

Supplement: Urbanization and humidity shape the intensity of influenza epidemics in U.S. cities [file Science-362-75-s002.zip › obs v sim comps all cities/548.jpg]

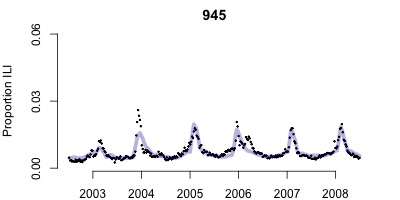

Supplement: Urbanization and humidity shape the intensity of influenza epidemics in U.S. cities [file Science-362-75-s002.zip › obs v sim comps all cities/945.jpg]

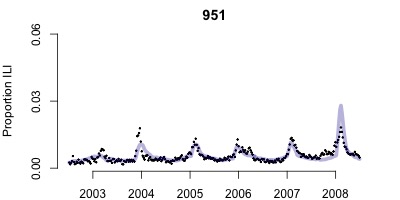

Supplement: Urbanization and humidity shape the intensity of influenza epidemics in U.S. cities [file Science-362-75-s002.zip › obs v sim comps all cities/951.jpg]

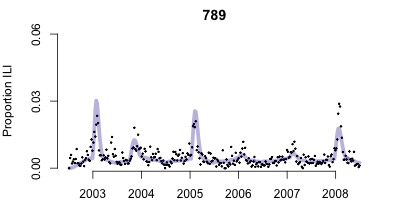

Supplement: Urbanization and humidity shape the intensity of influenza epidemics in U.S. cities [file Science-362-75-s002.zip › obs v sim comps all cities/789.jpg]

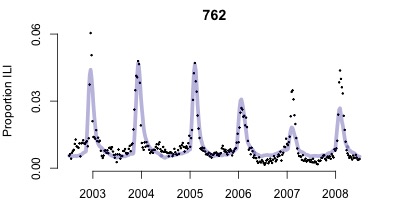

Supplement: Urbanization and humidity shape the intensity of influenza epidemics in U.S. cities [file Science-362-75-s002.zip › obs v sim comps all cities/762.jpg]

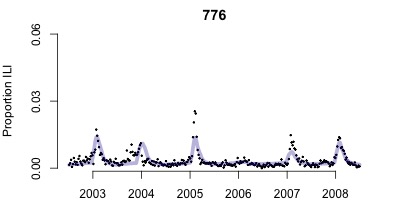

Supplement: Urbanization and humidity shape the intensity of influenza epidemics in U.S. cities [file Science-362-75-s002.zip › obs v sim comps all cities/776.jpg]

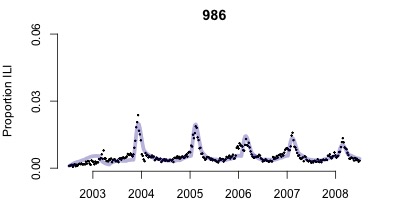

Supplement: Urbanization and humidity shape the intensity of influenza epidemics in U.S. cities [file Science-362-75-s002.zip › obs v sim comps all cities/986.jpg]

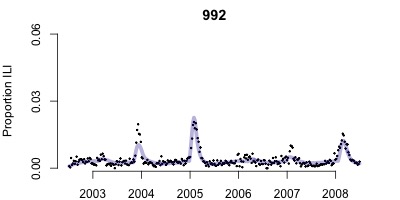

Supplement: Urbanization and humidity shape the intensity of influenza epidemics in U.S. cities [file Science-362-75-s002.zip › obs v sim comps all cities/992.jpg]

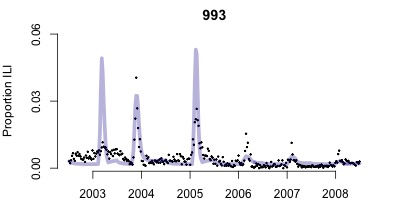

Supplement: Urbanization and humidity shape the intensity of influenza epidemics in U.S. cities [file Science-362-75-s002.zip › obs v sim comps all cities/993.jpg]

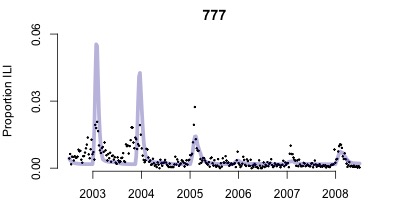

Supplement: Urbanization and humidity shape the intensity of influenza epidemics in U.S. cities [file Science-362-75-s002.zip › obs v sim comps all cities/777.jpg]

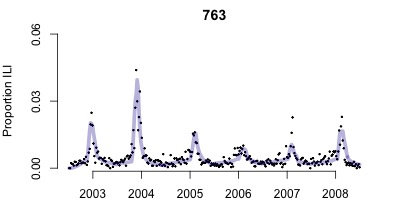

Supplement: Urbanization and humidity shape the intensity of influenza epidemics in U.S. cities [file Science-362-75-s002.zip › obs v sim comps all cities/763.jpg]

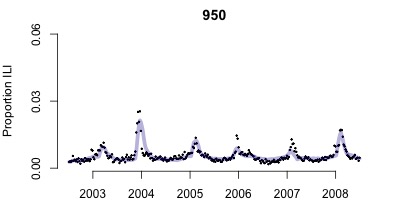

Supplement: Urbanization and humidity shape the intensity of influenza epidemics in U.S. cities [file Science-362-75-s002.zip › obs v sim comps all cities/950.jpg]

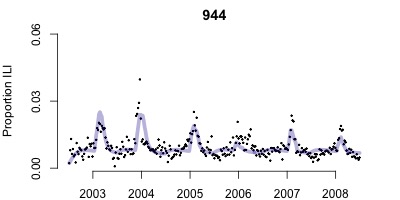

Supplement: Urbanization and humidity shape the intensity of influenza epidemics in U.S. cities [file Science-362-75-s002.zip › obs v sim comps all cities/944.jpg]

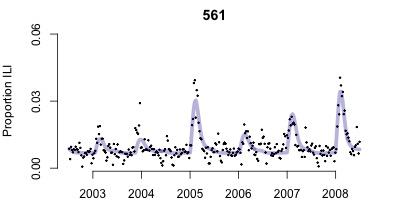

Supplement: Urbanization and humidity shape the intensity of influenza epidemics in U.S. cities [file Science-362-75-s002.zip › obs v sim comps all cities/561.jpg]

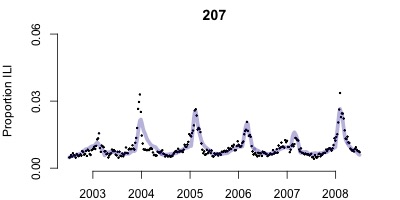

Supplement: Urbanization and humidity shape the intensity of influenza epidemics in U.S. cities [file Science-362-75-s002.zip › obs v sim comps all cities/207.jpg]

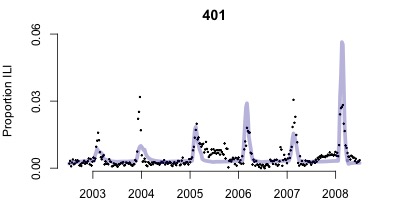

Supplement: Urbanization and humidity shape the intensity of influenza epidemics in U.S. cities [file Science-362-75-s002.zip › obs v sim comps all cities/401.jpg]

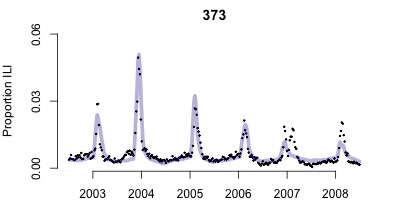

Supplement: Urbanization and humidity shape the intensity of influenza epidemics in U.S. cities [file Science-362-75-s002.zip › obs v sim comps all cities/373.jpg]
